# Thermodynamic controls of the Western Tibetan Vortex on Tibetan air temperature

**DOI:** 10.1007/s00382-019-04785-2

**Published:** 2019-05-03

**Authors:** Xiao-Feng Li, Hayley J. Fowler, Jingjing Yu, Nathan Forsythe, Stephen Blenkinsop, David Pritchard

**Affiliations:** School of Engineering, Newcastle University, Newcastle upon Tyne, NE1 7RU UK

**Keywords:** Western Tibetan Vortex, Karakoram vortex, Subtropical westerly Jet, Adiabatic heating, Thermal energy balance, Thermodynamic energy equation

## Abstract

The “Western Tibetan Vortex” (WTV)—also termed the Karakoram Vortex—dominates the middle-to-lower troposphere and the near-surface air temperature variability above the western Tibetan Plateau (TP). Here, we explore the thermodynamic mechanisms through which the WTV modulates air temperature over the western TP by diagnosing the three major terms of the thermodynamic energy equation—adiabatic heating, horizontal temperature advection, and diabatic heating—that maintain the atmospheric thermal balance. We composite these major terms to examine the differences between anti-cyclonic and cyclonic WTV events. Our theoretical approach demonstrates that adiabatic sinking-compression (rising-expansion) provides the overwhelming control on both the middle-to-lower tropospheric and lower stratospheric temperature increases (decreases) under anti-cyclonic (cyclonic) WTV conditions over the western TP high mountain area in all four seasons. This also explains the mechanisms behind the anomalous temperature “dipole” found between the mid-lower troposphere and lower stratosphere when the WTV was initially identified. Spatially, adiabatic heating effects are centred on the central western TP in summer and the south slope centring at 70°–80°E of the TP in other seasons. The other two terms, horizontal temperature advection and diabatic heating, have localized importance over the edges of the western TP. In a case study over the Karakoram area, we further demonstrate that adiabatic heating (rising-expanding-cooling/sinking-compressing-warming) is the dominant thermodynamic process controlling Karakoram air temperatures under WTV variability, except for at the very near surface in autumn and winter. Our analysis methods can be applied to investigate the thermodynamic processes of other atmospheric circulation systems or climate variability modes.

**Electronic supplementary material:**

The online version of this article (10.1007/s00382-019-04785-2) contains supplementary material, which is available to authorized users.

## Introduction

The “Western Tibetan Vortex” (WTV)—or “Karakoram Vortex”—has recently been recognized (Forsythe et al. 2017; Li et al. 2018; hereafter collectively cited as FL1718) as an anomalous large-scale deep vortex system which prevails over the western Tibetan Plateau (TP) in all four seasons, spanning 3–4 times the west–east breadth of the Indian Peninsula. Its intensity is measured by the Karakoram Zonal Index (KZI) (Forsythe et al. 2017); positive (negative) KZI values indicate an anomalous anti-cyclonic (cyclonic) WTV. The WTV provides a dominant driver (Li et al. 2018) of circulation variability over the western TP as it can explain over 50% variance of the western TP circulation on multiple levels throughout the troposphere. The influence of the WTV declines towards the eastern TP, resulting in a “west strong, east weak” spatial pattern in the variance of geopotential height (HGT) over the whole TP in most seasons. The WTV also influences middle-to-lower tropospheric and near-surface air temperature (FL1718), precipitation (Li et al. 2018) and ozone (Li et al. 2018) across the western TP. Specifically, FL1718 found that the WTV explains over half (> 50%) of the summertime air temperature variance in the middle-to-lower troposphere and at the near-surface over the Karakoram sub-region of the western TP. In quantitative terms, one standard deviation change of the summertime KZI causes ~  0.74 K (Forsythe et al. 2017) change in the station observed 2 m air temperature ($T_{2m}$) over the Karakoram. The cooling of 2 m air temperature over the western TP caused by the downward trend of the WTV since 1960s provides a mechanism (Forsythe et al. 2017) to understand the stability of glaciers and the reduction in river flows over the western TP in summer seen in recent decades, e.g. the “Karakoram anomaly” (e.g., Hewitt 2005; Gardelle et al. 2012; Jacob et al. 2012; Kääb et al. 2012; Pratap et al. 2016; Bolch et al. 2017; Zhou et al. 2017). Therefore, the WTV is an important circulation system driving climate variability over the TP.

**Fig. 1:**
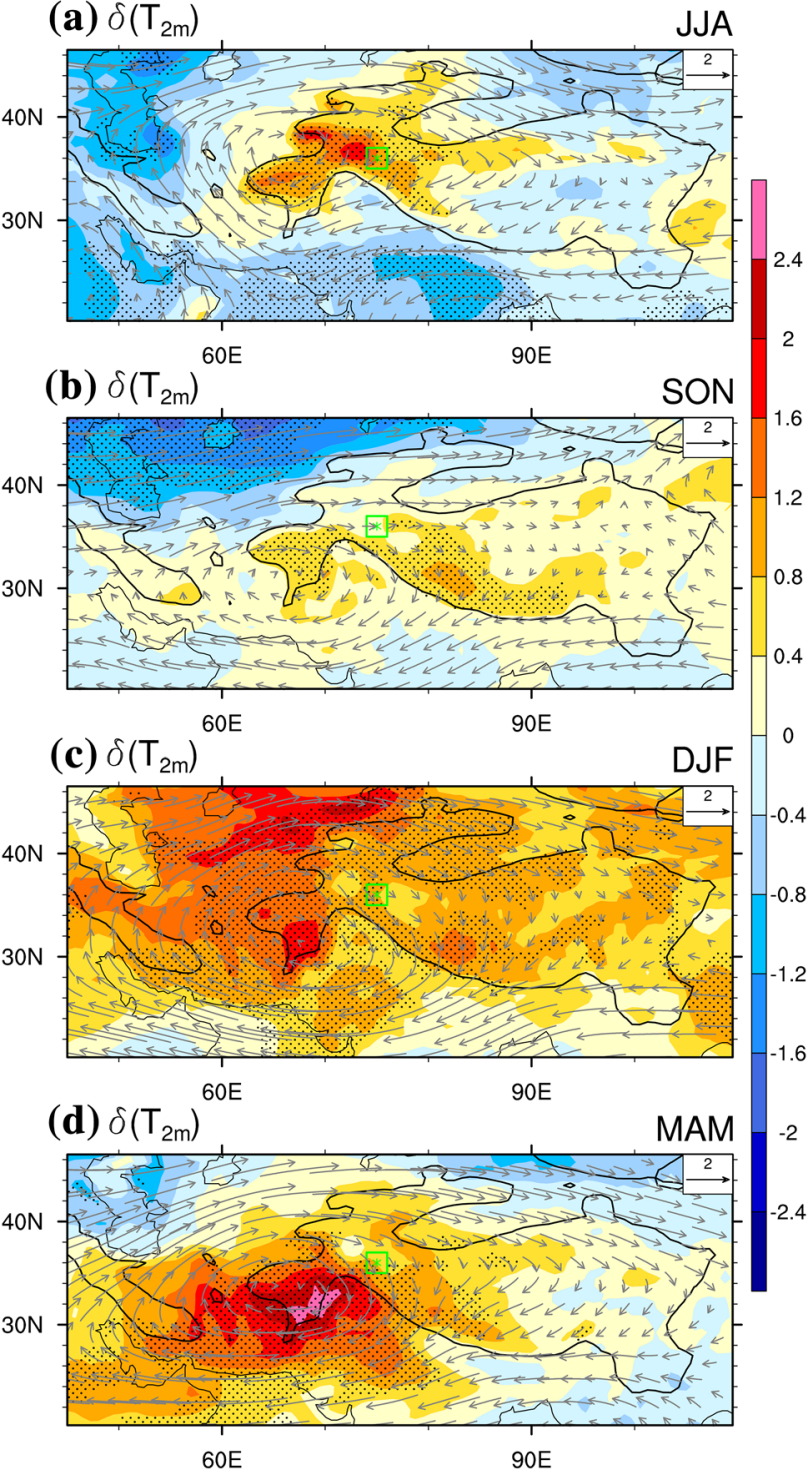
The difference in 2 m air temperature ($\delta \left( {T_{2m} } \right)$, color shading, K) and 500-hPa wind vectors (grey vectors, m s^−1^) between positive and negative KZI events (positive minus negative phase) in **a** summer (JJA), **b** autumn (SON), **c** winter (DJF) and **d** spring (MAM) for 1979–2016. The stippling denotes significance above the 0.10 level, after taking account of the efficient number of degrees of freedom (Zar 1984; Li et al. 2013). Wind vector length scale is in the upper right corner of panels. The green star denotes the central position (36°N, 75°E) of the Karakoram focus area, the green square denotes area of 35–37°N, 74–76°E. The bold-black-outline denotes topography above 1500 m

Generally, an anomalous temperature “dipole” between the middle-to-lower troposphere and the lower stratosphere over the western TP is observed when the KZI is strongly positive (negative), the WTV is a deep anti-cyclone (cyclone) (FL1718). As a result, the lower stratosphere gets colder (warmer) while the middle-to-lower troposphere gets warmer (colder). Uniquely, in the middle-to-lower troposphere, the WTV manifests as a “warm high/cold low” structure (FL1718). As verified in Fig. 1, the composite of positive (negative) KZI events (methods see Sect. 2.3) in the horizontal wind field is characterized as an anti-cyclonic (cyclonic) structure over the near surface of the TP (also see Figures from FL1718). There is thus substantial rotation/vorticity around the high (low) pressure center of the WTV. This drives atmospheric mass and energy exchange between the western TP and surrounding areas. The temperature anomalies associated with WTV variability in the middle-to-lower troposphere also extend downward to the surface level of the TP, evidenced as $T_{2m}$ over the central and western TP is also significantly increased (decreased) when the WTV is anti-cyclonic (cyclonic). The geographic center of these responses are over the west tail of the TP, although there are seasonal displacements.

The warm high (cold low) structure of the WTV in the middle-to-lower troposphere (FL1718) means that air temperature variability over the western TP is induced by the WTV variability, not the opposite, because a heat-induced atmospheric vortex system is usually a warm low or cold high structure in the mid-lower troposphere (e.g., Holton 2004; Poulidis et al. 2016). FL1718 have previously suggested that the WTV exerts its influence on the middle-to-lower tropospheric and the surface air temperature over the Karakoram by adiabatic rising-expanding-cooling/sinking-compressing-warming. By compositing the anomalous circulations in positive and negative phases of the WTV, Forsythe et al. (2017) found that strong downward (upward) vertical wind anomalies usually occur in areas with the highest (lowest) temperatures in positive (negative) KZI events in both winter and summer. This describes a typical adiabatic thermal process associated with WTV variability. As the lower layer of the atmosphere usually has a greater density than the upper layer, in the positive (negative) phase of the WTV, the anomalous downward (upward) motion means the anomalous atmospheric mass parcels penetrate downward (upward) into the denser (lighter) pressure levels. In the denser (lighter) pressure levels, the volume of the atmospheric mass parcels are naturally compressed (expanded), causing the temperature in the atmospheric mass parcels to increase (decrease) adiabatically. Li et al. (2018) documented similar significant adiabatic rising-expanding-cooling/sinking-compressing-warming processes in the core mass volume of the WTV in all four seasons, verifying the results of Forsythe et al. (2017). In addition, the climatological mean wind speed at the near-surface of the western TP is much lower than for the surrounding areas (see Fig. 1S in Supplementary Information). This suggests relatively limited horizontal mass and energy exchanges between the western TP and adjacent regions. This enhances the relative dominance of the impact of the WTV on $T_{2m}$ from adiabatic rising-expanding-cooling/sinking-compressing-warming processes.

However, the emphasis on adiabatic rising-expanding-cooling/sinking-compressing-warming alone might oversimplify the thermodynamic mechanisms associated with WTV variability. Because the WTV is seasonally partly coupled (Forsythe et al. 2017) with the South Asia Summer Monsoon (e.g., Lau et al. 2012), the behavior of the WTV in summer is also likely influenced by diabatic heating and the thermally-driven horizontal temperature advection through the monsoon circulation; not evaluated in previous analyses (FL1718). Therefore, although it seems likely that adiabatic rising-expanding-cooling/sinking-compressing-warming processes are the dominant control on temperature changes associated with WTV variability based on observations from FL1718, the theoretical verification has not yet been undertaken. Correspondingly, the relative contributions of this and other thermal processes, specifically diabatic heating and horizontal temperature advection, have not yet been quantified. Moreover, the thermodynamic mechanisms through which the WTV modulates temperature variability in the lower stratosphere have not yet been confirmed. Similarly, the mechanisms by which the WTV causes the anomalous temperature “dipole” between the middle-to-lower troposphere and lower stratosphere above the western TP have not yet been identified. Recognizing these knowledge gaps, we here employ the thermodynamic energy equation (TEE: Holton 2004) to quantify the relative contributions of various components of heating in driving the observed temperature anomalies related to WTV variability over the western TP. This serves to clarify the major and minor thermodynamic mechanisms through which the WTV exerts its influence on air temperature variability in both the middle-to-lower troposphere and the lower stratosphere over the western TP high mountain area.

The remainder of this paper is organized as follows. Section 2 presents data and methods. In this section, we summarize the derivations of the thermal energy equation (TEE) and the thermal balance for seasonal mean circulations based on previous studies (e.g., Holton 2004; Duan and Wu 2006; Yu 2009; Yu et al. 2011a, b), and defining the formulas of the composited differences of the TEE terms which are subsequently evaluated in this study. Finally, the formula of the law of conservation between the composited differences of the three major TEE terms under the thermal equilibrium or the the modified thermal equilibrium formula (see Eq. 11) is derived for the convenience of the analysis in this study. In Sect. 3, the basic features of circulation variability related to the thermodynamic processes over the western TP associated with the WTV behavior are studied. In Sect. 4, we examine the general contributions of the TEE terms to tropospheric temperature changes by examining the area-averaged differences of the three major TEE terms over the entire western TP and the vertical profiles over the central western TP throughout the year. In Sect. 5, we examine the horizontal distributions of the contributions from the TEE terms in the middle-to-lower troposphere season by season. In Sect. 6, we investigate the contributions of the TEE terms to lower stratospheric temperature changes. Then, in Sect. 7 we present a regional case study of the relative contributions of these thermodynamic terms on both the mid-low tropospheric and lower stratospheric temperature changes under WTV variability over the Karakoram. Finally, discussion of the results and our conclusions are presented in Sect. 8.

## Data and methods

### Data

We use monthly mean values from the ERA-Interim reanalysis (Dee et al. 2011) (http://apps.ecmwf.int/datasets/) to perform our analyses, because it not only has high horizontal and vertical resolutions, but also was demonstrated to have a better performance among the existing reanalysis datasets in depicting the thermal conditions over the western TP high mountain areas in Forsythe et al. (2017). The spatial resolution is 0.75° × 0.75° in the horizontal direction and 26 levels in the vertical direction. The variables used from ERA-Interim include: the zonal wind velocity (m s^−1^); the meridional wind velocity (m s^−1^); the vertical wind velocity (Pa s^−1^) represented by the reversed Omega (so a positive value of the vertical wind velocity denotes upward motion, and vice versa); the geopotential height (HGT, m^2^ s^−2^ or gpm); air temperature (K) on multiple isobaric levels; the surface air temperature (K) at 2 m elevation; and the total column ozone (10^−2^ g m^−2^). The KZI was originally defined by Forsythe et al. (2017) based on winter and summer mean zonal wind, and then developed as a standardized monthly index with continuous values running across the whole year. Here, the monthly KZI is calculated according to the method in Li et al. (2018).

We focus on the 1979–2016 period, but define the circulation anomaly as the departure from the climatological mean circulation during the reference period of 1979–2010 for consistency with previous studies (Forsythe et al. 2017; Li et al. 2018). In calculating the monthly anomaly time series, the climatological mean is assessed for each month of the annual cycle. Following Zar (1984) and Li et al. (2013), the efficient number of degrees of freedom are taken into account in the 2-tailed *student’s t test* (von Storch and Zwiers 2002) of the correlations.

### Thermal energy equation and thermal equilibrium

The atmospheric TEE (Holton 2004) is widely used to diagnose the atmospheric thermal balance, including the atmospheric thermal balance over the Tibetan Plateau (e.g., Duan and Wu 2006; Yu 2009; Yu et al. 2011a, b). The atmospheric thermal energy equation is derived from the first law of thermodynamics (Holton 2004), which is written as:

1 $$dI = dQ - dW.$$

where $dI$ is the change of internal energy (due to the kinetic energy of the individual molecules, which can be measured by temperature, $T$), $dQ$ is the change of diabatic heating (due to sensible heating, radiative heating, latent heating, etc., which are effected though conduction, radiation and release etc.), and $dW$ is the work done by the parcel (due to macroscopic motion, i.e. expansion or compression). It shows that the internal energy change of an atmospheric mass parcel is determined by the difference between the diabatic heating received by the atmospheric mass parcel and the work done by the expanding parcel (Holton 2004).

For an expanding or compressing atmospheric parcel, the pressure is a force per unit area doing work, which is given by the product of force and velocity vectors (Holton 2004). Thereby, pressure change is considered in the atmospheric energy change. In addition, the principle of energy conservation is applied to the Lagrangian atmospheric mass parcel (but the effects of molecular viscosity are neglected) (Holton 2004). Then, the first law of thermodynamics is changed into the TEE [see Eq. (2)], in which the temporal changes of the internal energy of the atmospheric mass parcel in the Lagrangian system is written into the total derivative form (Holton 2004) as follows:

2 $$\frac{dT}{dt} = \frac{1}{{C_{p} }}\dot{Q} + \frac{1}{{C_{p} \rho }}\frac{dp}{dt},$$

where $T$ is the temperature measuring the internal energy, $t$ is the time, $p$ is the atmospheric pressure, $\rho$ is the atmospheric density, $C_{p}$ is the specific heat capacity of the atmosphere at constant pressure, $\dot{Q}$ is the rate of heating per unit mass due to radiation, conduction, latent heat release, etc. The term $\frac{dT}{dt}$ is the instantaneous temporal change of temperature in the atmospheric parcel, the term $\frac{1}{{C_{p} \rho }}\frac{dp}{dt}$ is the thermal chan in the atmospheric parcel caused by the temporal change in adiabatic compression, and the term $\frac{1}{{C_{p} }}\dot{Q}$ the thermal change in atmospheric parcel caused by the rate of diabatic heating. Thus, temporal changes of temperature (of the atmospheric mass parcel) are determined by the temporal changes of the adiabatic cpression and the rate of the diabatic heating.

In an isobaric coordinate system, $\frac{dp}{dt} = \omega$, where $\omega$ is the vertical velocity. Besides, $\rho = \frac{p}{RT}$ according to the atmospheric continuity equation (Holton 2004). Equation (2) can thereby be changed into:

3 $$\frac{dT}{dt} = \frac{1}{{C_{p} }}\dot{Q} + k\frac{T}{p}\omega .$$

where $k = \frac{{R_{d} }}{{C_{p} }}$, $R_{d}$ is a gas constant. To proceed, the total derivation in Eq. (3) can be expanded into a partial derivation, which converts the temporal changes of temperature of atmospheric mass parcel in the Lagrangian frame into the temporal changes of it in the Eulerian frame in partial derivative form on an isobaric surface (e.g., Holton 2004; Duan and Wu 2006; Yu 2009; Yu et al. 2011a, b):

4 $$\frac{\partial T}{\partial t} = - V \cdot \nabla_{p} T - \omega \left( {\frac{\partial T}{\partial p} - k\frac{T}{p}} \right) + \frac{1}{{C_{p} }}\dot{Q}$$

where $V$ is the horizontal wind vector that is composed of the zonal wind and meridional wind components. The term $- V \cdot \nabla_{p} T$ is the horizontal temperature advection on an isobaric surface. The term $- \omega \left( {\frac{\partial T}{\partial p} - k\frac{T}{p}} \right)$ is the vertical temperature advection, where $\frac{\partial T}{\partial p} - k\frac{T}{p}$ is the static stability of the atmosphere. Then, the Potential Temperature (Holton 2004), ${{\theta }}$, is introduced, which is defined as:

5 $$\theta = T\left( {\frac{p}{{p_{s} }}} \right)^{{\frac{{R_{d} }}{{C_{p} }}}} ,$$

$\theta$ is the temperature that a parcel of dry air at pressure $p$ and temperature $T$ would have if it were expanded or compressed adiabatically to a standard pressure $p_{s}$ (usually taken as 1000 hPa) (Holton 2004). By introducing $\theta$, the vertical advection term in Eq. (4) is simplified into a single term, $- \omega \left( {\frac{p}{{p_{s} }}} \right)^{k} \frac{\partial \theta }{\partial p}$ (e.g., Holton 2004; Duan and Wu 2006; Yu 2009; Yu et al. 2011a, b) and Eq. (4) is written as:

6 $$\frac{\partial T}{\partial t} = - V \cdot \nabla_{p} T - \omega \left( {\frac{p}{{p_{s} }}} \right)^{k} \frac{\partial \theta }{\partial p} + \frac{{\dot{Q}}}{{C_{p} }}$$

Equation (6) is the conventional form of the TEE in the isobaric coordinate system (e.g., Holton 2004; Duan and Wu 2006; Yu 2009; Yu et al. 2011a, b). It constrains the instantaneous thermal energy changes of the atmosphere on isobaric surfaces in the Eulerian frame.

For temporal mean atmosphere, the TEE is changed (e.g., Holton 2004; Duan and Wu 2006; Yu 2009; Yu et al. 2011a, b) from Eq. (6) by applying the formula $\overline{AB} = \bar{A} \cdot \bar{B} + AB$, which is written as:

7 $$\frac{{\partial \bar{T}}}{\partial t} = - \bar{V} \cdot \le \nabla_{p} \bar{T} - \nabla_{p} \cdot \left( {VT} \right) - \left( {\frac{p}{{p_{s} }}} \right)^{k} \bar{\omega }\frac{{\partial \bar{\theta }}}{\partial p} - \left( {\frac{p}{{p_{s} }}} \right)^{k} \frac{\partial }{\partial p}\left( {\bar{\omega }\bar{\theta }} \right) + \frac{{\bar{\dot{Q}}}}{{C_{p} }} ,$$

where “–” is the temporal mean, “′” is deviation against the temporal mean. For the temporal mean over a longer time span (e.g., a day, month or season), the temporal variation of the internal energy ($\frac{{\partial \bar{T}}}{\partial t}$) is so small that can be neglected (e.g., Duan and Wu 2006). The transient disturbances (the terms $- \nabla_{p} \cdot \overline{{\left( {VT} \right)}}$ and $- \left( {\frac{p}{{p_{s} }}} \right)\frac{\partial }{\partial p}\overline{(\omega \theta )}$ are relatively not important in data with big time-steps (e.g., Duan and Wu 2006). Therefore, the thermal equilibrium (e.g., Holton 2004; Duan and Wu 2006; Yu 2009; Yu et al. 2011a, b) for the temporal mean atmosphere can be approximated as:

8 $$0 \approx - \left( {\frac{p}{{p_{s} }}} \right)^{k} \bar{\omega }\frac{{\partial \bar{\theta }}}{\partial p} - \bar{V} \cdot \nabla_{p} \bar{T} + \frac{{\bar{\dot{Q}}}}{{C_{p} }}.$$

This means that the thermal balance for the temporal mean atmosphere over a longer time span is mainly maintained between three major terms: the adiabatic heating (ADH) term ($- \left( {\frac{p}{{p_{s} }}} \right)^{k} \bar{\omega }\frac{{\partial \bar{\theta }}}{\partial p}$) that represents the adiabatic sinking (compressing) or rising (expanding) process, the horizontal temperature advection (HTAD) term ($- \bar{V} \cdot \nabla_{p} \bar{T}$) that represents the effects of the warm or cold currents, and the diabatic heating (DH) term ($\frac{{\bar{\dot{Q}}}}{{C_{p} }}$) that represents the net effects of the sensible heating conduction, radiative heating, latent heating release, and etc. In practice, the DH term ($\frac{{\bar{\dot{Q}}}}{{C_{p} }}$) is calculated using other two terms (Duan and Wu 2006; Yu 2009; Yu et al. 2011a, b). In addition, it should be noted that some studies (e.g., Duan and Wu 2006) also decomposed the HTAD term ($- \bar{V} \cdot \nabla_{p} \bar{T}$) into two components, the zonal temperature advection and the meridional temperature advection, but these two terms cancel each other heavily over the TP area (e.g., Duan and Wu 2006), so we use just one vector term to measure the net horizontal temperature advection in this study, following Yu (2009) and Yu et al. (2011a, b).

### Compositing of the major thermodynamic terms

To investigate the relative contributions among the major terms of the TEE to the temperature changes over the western TP related to WTV variability, we composite the three major terms of Eq. (8), including the ADH term, the HTAD term, and the DH term. Through the compositing of these terms and the air temperature in different seasons according to the negative and positive KZI events, we can examine their relative contributions to air temperature changes over the western TP by using the differences of the three terms between positive and negative KZI events. A similar method has been used in previous studies over the TP (Duan and Wu 2006; Yu 2009; Yu et al. 2011a, b).

We define a positive (negative) KZI event in a season (i.e., winter, spring, summer or autumn) as one where the seasonal mean KZI is above (below) 0.5 (− 0.5) times its standard deviation for that season for all years in the period of 1979–2016, as defined in FL1718. As a result, there are enough samples sizes for both the positive and negative KZI events in four seasons, as shown in Table 1. The slightly different number of events in different seasons is caused by the slightly different shapes of the probability distributions of the KZI in different seasons. Then, the differences of the three terms (in units of K day^−1^) between the positive and negative KZI events (i.e., composited terms in positive KZI events minus those in negative KZI events) are analyzed, respectively. Denoting

9 $$\delta \left( {} \right) = \left( {} \right)_{PKZI} - \left( {} \right)_{NKZI} .$$

where subscripts PKZI and NKZI indice the mean values of a variable or a TEE term in positive and negative KZI events, $\delta \left( {} \right)$ represents the composited difference of the variable or the TEE term. The composited differences of the terms in Eq. (8) can be denoted as

10 $$\left\{ {\begin{array}{*{20}ll} {\delta \left( {ADH} \right) = \delta \left( { - \left( {\frac{p}{{p_{s} }}} \right)^{k} \bar{\omega }\frac{{\partial \bar{\theta }}}{\partial p}} \right)} \\ {\delta \left( {HTAD} \right) = \delta \left( { - \bar{V} \cdot \le \nabla_{p} \bar{T}} \right)} \\ {\delta \left( {DH} \right) = \delta \left( {\frac{{\bar{\dot{Q}}}}{{C_{p} }}} \right)} \\ \end{array} } \right. .$$

where $\delta \left( {ADH} \right)$, $\delta \left( {HTAD} \right)$ and. indicate the composited differences of the three TEE major terms. According to Eq. (8) and (11), we can also derive the conservation law of the composited differences of the three major TEE terms under thermal equilibrium:

**Table 1:** Number of positive and negative KZI events for the period 1979–2016

	Number of positive events	Number of negative events
Summer (JJA)	12	12
Autumn (SON)	14	14
Winter (DJF)	10	9
Spring (MAM)	16	13

For each season, a positive (negative) KZI event is defined as the season in a year when the KZI is bigger (smaller) than 0.5 (− 0.5) times the standard deviation of the KZI in that season during the period 1979–2016

11 $$0 \approx \delta \left( {ADH} \right) + \delta \left( {HTAD} \right) + \delta \left( {DH} \right).$$

Here, we also call Eq. (11) as the modified thermal equilibrium formula (MTEF). This means that the composited differences of three major TEE terms between positive and negative KZI events are in equilibrium, i.e. they balance/counteract each other. This is in accordance with the law of conservation energy as represented in the composited differences of three major TEE terms. Physically, the $\delta \left( {ADH} \right) > 0$ ($\delta \left( {ADH} \right) < 0$ ans the effect of the adiabatic sinking compressing (rising expanding) occurs during the positive KZI events relative to the negative KZI events; the $\delta \left( {HTAD} \right) > 0$ ($\delta \left( {HTAD} \right) < 0$) indicates the effect of the warm (cold) current that brings in the warmer (colder) air from neighbouring areas; and the $\delta \left( {DH} \right) > 0$ ($\delta \left( {DH} \right) < 0$) means the increased (decreased) net effects in sensible heating conduction, radiation, latent heating release and etc. In summary the positive (negative) composited difference by the three individual TEE terms intends to cause increase (decrease) in the local temperature.

It has been demonstrated (FL1718 at the temperature in the mid-lower troposphere ($T$) and 2 m surface air ($T_{2m}$) over the western TP increases from the negative to the positive KZI events, i.e. $\delta \left( {T_{2m} } \right) > 0$ and $\delta \left( T \right) > 0$. The positive (negative) composited difference of a TEE term in the mid-to-lower troposphere over the western Tmeans this TEE term is positively (negatively) contributing to the temperature increases. In contrast, the air temperature in the lower stratosphere decreases during positive KZI events relative to the negative KZI events (FL1718), i.e. $\delta \left( T \right) < 0$. The negative (positive) composited difference of a TEE term over the western TP means this TEE term is negatively (positively) contributing to the temperature decreases in the lower stratosphere. Anyhow, the thermodynamic process(es) represented by the TEE term(s) with positive contribution(s) can be treated as the major driving mechanism(s) through which the WTV causes the temperature increase over the western TP. For multiple thermodynamic processes with positive (or negative) contributions, their relative contributions are measured by the ratio of the absolute values of the composited differences of the corresponding TEE terms.

We determine the significance level of the differences based on the 2-tailed student’s *t* test (von Storch and Zwiers 2002) after the effective sample sizes are estimated (Zwiers and von Storch 1995).

### Western Tibetan circulation chang under the WTV variability

To investigate the thermal processes over the western TP related to the WTV variability, it is necessary to study the general features of the circulations over the western TP and the basic characteristics of the WTV that are connected to thermodynamic processes.

### Tropospheric and 2 m surface air temperature changes

**Fig. 2:**
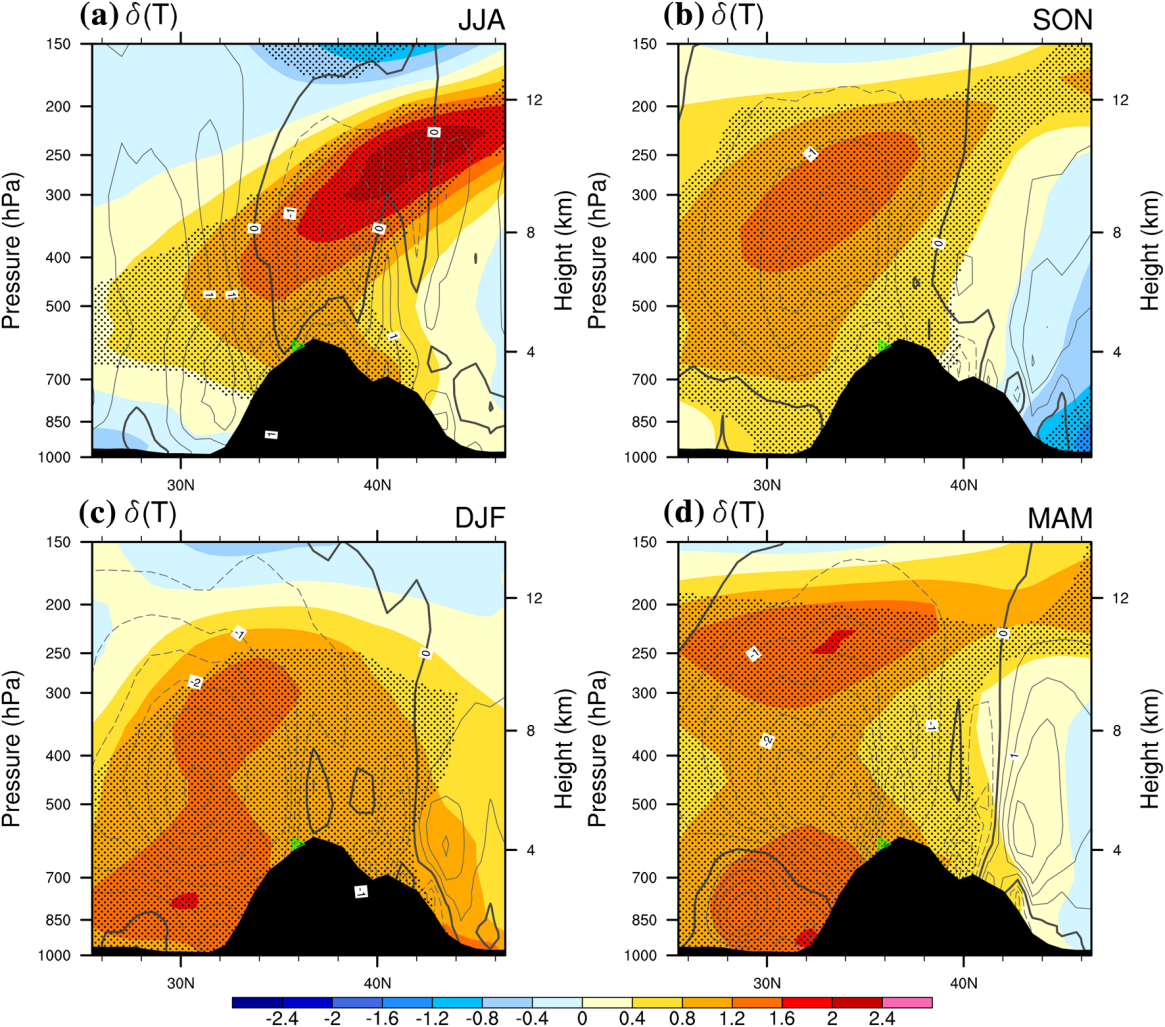
The composited difference in air temperature ($\delta \left( T \right)$, color shading, K) and vertical wind velocity ($\delta \left( w \right)$, grey contours, 10^−1^ Pa s^−1)^ between positive and negative KZI events (positive minus negative phase) along a latitudinal profile (across 70°E–80°E) in **a** summer (JJA), **b** autumn (SON), **c** winter (DJF) and d) spring (MAM) for 1979–2016. The stippling denotes significance of air temperature above the 0.10 level, after taking account of the efficient number of degrees of freedom (Zar 1984; Li et al. 2013). The solid, dashed and bold solid grey contour lines denote the positive, negative and zero values of $\delta \left( w \right)$, the positive (negative) $\delta \left( w \right)$ denotes anomalous upward (downward) motion. Black shaded area denotes the topography. The green triangle denotes the central Karakoram (36°N, 75°E)

It should be emphasized that, in responding to WTV variability, the 2 m surface air temperature ($T_{2m}$) anomalies are linked to the larger scale of air temperature changes extending downward from the middle-to-high troposphere. The color shading in Fig. 2 indicates that in summer (Fig. 2a) and autumn (Fig. 2b) the greatest positive response of air temperature ($\delta \left( T \right) > 0$, $\delta \left( T \right)$ is the difference of the composited $T$ in positive KZI events minus that in negative KZI events; see Sect. 2 for more details) to WTV variability above the central western TP (70°E–80°E) is located in the middle-to-high troposphere at around the 300–250 hPa level. This warm anomaly extends downward to the near surface of the western TP. This is also verified in Table 2, in summer (autumn), the averaged response of air temperature over the western TP high mountain areas at the 300-hPa level is 0.77 (0.94) K, which drops to 0.55 (0.55) K at 500-hPa, then dropping further to 0.50 (0.33) K at the near-surface (2 m). This demonstrates the downward extension of the temperature response in summer and autumn from the middle-high troposphere to the near-surface.

**Table 2:** Area-averaged differences of the TEE terms (K Day^−1^) and air temperature (K) over multiple levels between positive and negative KZI events over the western TP (24.75°N–44.5°N, 60°E–90°E) high mountain area (altitude above 1500 m) in 1979–2016

		500-hPa	300-hPa	70-hPa
Summer (JJA)	$\delta \left( {ADH} \right)$	**0.0365**	**0.060**	− **0.562**
	$\delta \left( {HTAD} \right)$	− 0.0144	− 0.069	0.493
	$\delta \left( {DH} \right)$	− 0.0221	**0.009**	0.069
	$\delta \left( T \right)$	0.55	0.77	− 0.99
	$\delta \left( {T_{2m} } \right)$		0.50	
Autumn (SON)	$\delta \left( {ADH} \right)$	**0.119**	**0.34**	− **0.075**
	$\delta \left( {HTAD} \right)$			0.024
	$\delta \left( {DH} \right)$			0.051
	$\delta \left( T \right)$	0.55	94	− 1.02
	$\delta \left( {T_{2m} } \right)$		33	
Winter (DJF)	$\delta \left( {ADH} \right)$	**0.27**	**0.49**	− **0.512**
	$\delta \left( {HTAD} \right)$	− 0.10	− 0.43	0.477
	$\delta \left( {DH} \right)$	− 0.17	− 0.06	0.035
	$\delta \left( T \right)$	0.95	1.00	− 1.95
	$\delta \left( {T_{2m} } \right)$		1.07	
Spring (MAM)	$\delta \left( {ADH} \right)$	**0.19**	**0.34**	− **0.190**
	$\delta \left( {HTAD} \right)$			0.194
	$\delta \left( {DH} \right)$			− **0.04**
	$\delta \left( T \right)$	0.63	74	− 1.52
	$\delta \left( {T_{2m} } \right)$		81	

The western TP high mountain area refers to the area with altitude above 1500 m within the area of 24.75°N–44.5°N, 60°E–90°E. The difference of each term is defined as the average for positive KZI events minus that for negative KZI events, which represents the contribution of the term to temperature increases over the western TP related to positive W. Here, the average of the differences of the terms over the western TP high mountain area is used to represent their mean contributions to temperature changes over the western TP. Values below 1500 m are masked out before the averaging. Negative (positive) values at 70 hPa (500 and 300 hPa) are shown in bold, which positively contributing to the lower stratospheric (tropospheric) temperature changes

In winter (Fig. 2c) and spring (gure 2d) the strongest positive response of air temperature ($\delta \left( T \right) > 0$) are also located at the middle-high troposphere at around 300–250 hPa; however, there are additional strong responses of air temperature found at the ~ 850 hPa level. Similarly, Table 2 indicates that in winter (spring) the averaged response of air temperature over the western TP high mountain area at the 300-hPa level is 1.0 (0.74) K, which drops to 0.95 (0.63) K at the 500-hPa level, then rises to 1.07 (0.81) K at the near-surface (2 m). The decrease from 300-hPa to 500-hPa verifies the downward extension of the temperature response from the middle-high troposphere to the mid-troposphere; while increase in temperature response from 500-hPa to the near-surface is consistent with the responses of the vertical temperature profile over the western TP documented by FL1718, who found the WTV has a deeper vertical structure in circulations, i.e. there are extra temperature change centres existing at the lower troposphere apart from the centres at the higher troposphere in winter and spring. As verified in Fig. 2c, d, in winter and spring, the strongest positive response of air temperature ($\delta \left( T \right) > 0$) are located at the higher troposphere at around 300–250 hPa; however, there are additional strong responses of air temperature found below the ~ 500 hPa level, which is mainly due to the strong adiabatic heating effects in middle-lower troposphere over the south slopes of the western TP, as demonstrated in Fig. 6d, g. But the complex radiation balance and surface turbulence processes in boundary layer (e.g., Oke 2002; Pritchard et al. 2019) can not be ruled out in contributing to this. In short, the increasing temperature response from 500-hPa to the 2 m near-surface in winter and spring is consistent with the vertical temperature response profile above the western TP, both the WTV-induced temperature profile in the lower troposphere and the complex surface processes could contribute to it.

Basically, the $T_{2m}$ responses over the western TP is a cross-section/profile of the air temperature change among multiple levels in the middle-to-lower troposphere related to the WTV variability. Thus, the $T_{2m}$ changes over high mountain areas of western TP are closely related to air temperature changes at 500 hPa that is the near-surface level of the western TP. Meanwhile $T_{2m}$ responses at the margins of the western TP and the neighbouring plain areas are closely related to air temperature changes at lower levels, with some minor effects due to the rugged TP topography. In others words, turbulent momentum and heat transport induced by surface roughness and other near-surface processes, e.g. radiation balance, in boundary layer (e.g., Oke 2002; Pritchard et al. 2019) could either dissipate or amplify the signal at the near-surface. Nevertheless, the $T_{2m}$ response to WTV variability is consistent with the response of the air temperature in the wider troposphere above the western TP.

### Vertical velocity anomalies

**Fig. 3:**
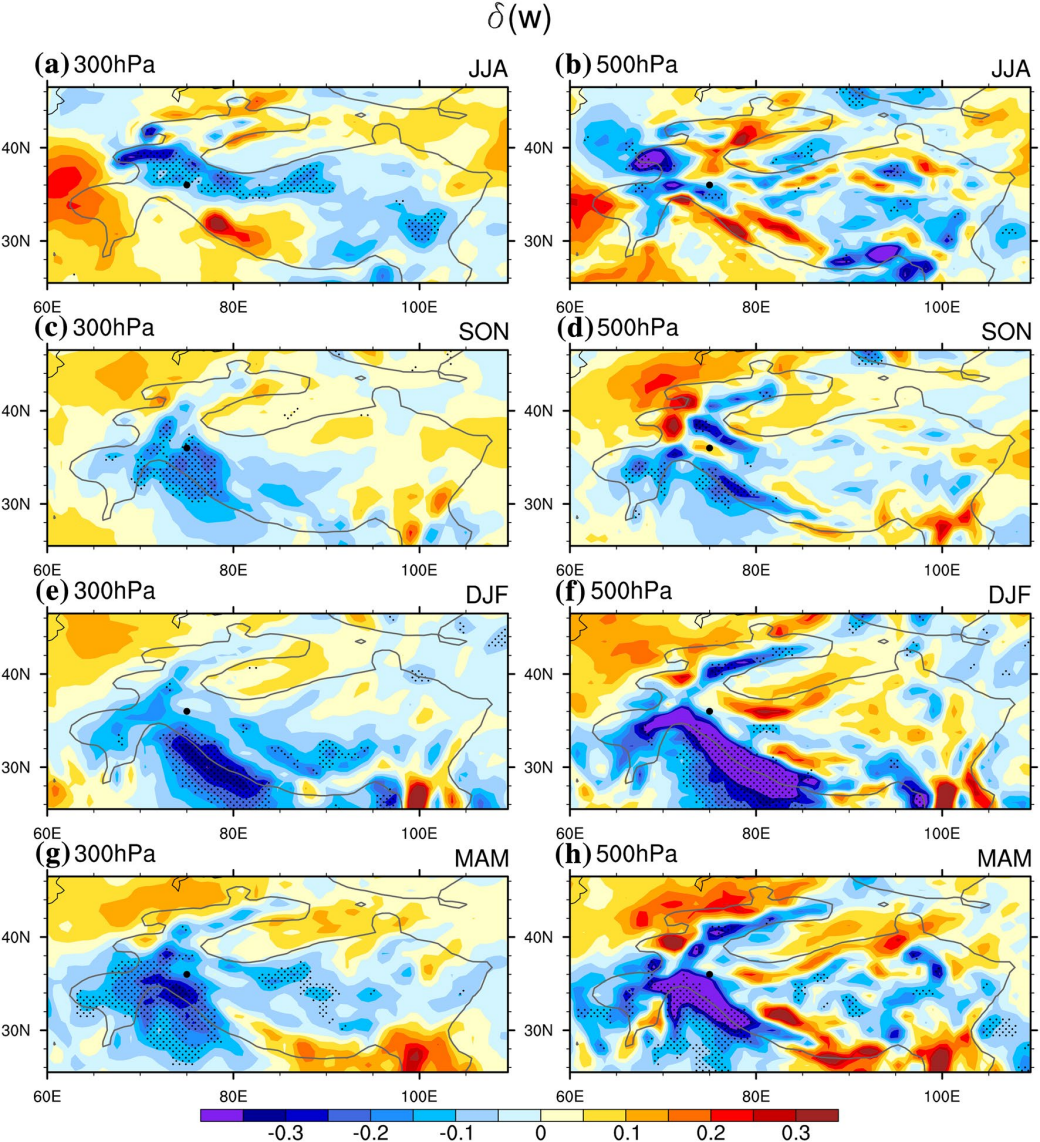
Differences in the vertical velocity ($\delta \left( w \right),$ color shading, 10^−1^ Pa s^−1^, the positive value denotes anomalous upward motion, and vice ver) bween positive and negative KZI events (positive minus negative phase) at multiple levels above the Western Tibetan Plateau for the period 1979–2016. **a**, **b** Are for summer (JJA), **c**, **d** are for autumn (SON), **e**, **f** are for winter (DJF), **g**, **h** are for spring (MAM). Left panels: **a**, **c**, **e**, **g** are for 300-hPa level, right panels: **b**, **d**, **f, h** are for 500-hPa level. Significant correlations above the 0.05 level are stippled, after taking account of the efficient number of degrees of freedom (Zar 1984; Li et al. 2013). Bold black dot denotes the central position (36°N, 75°E) of the Karakoram focus area. Bold-black-outline denotes topography abo 1500 m

**Fig. 4:**
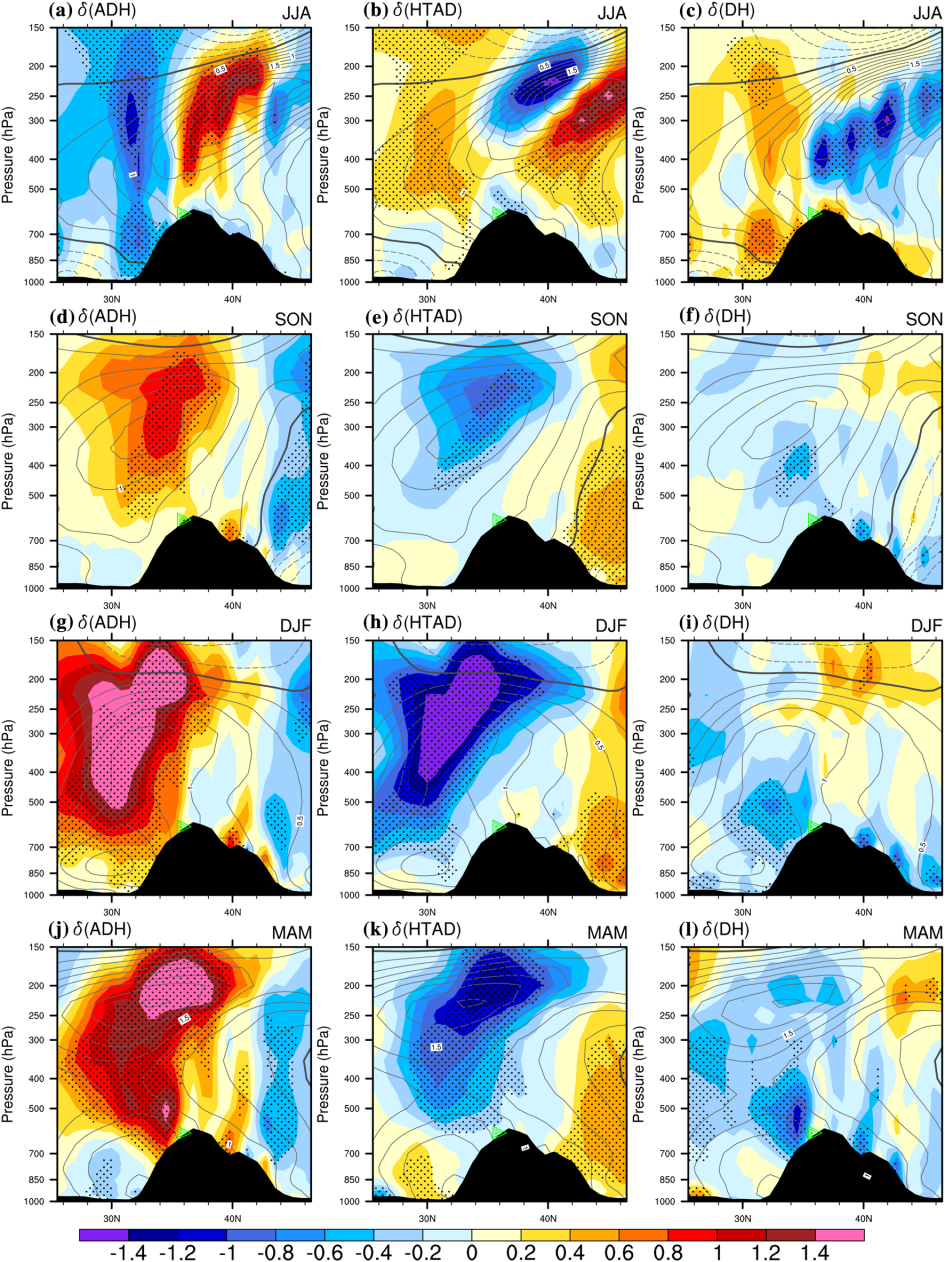
The difference of the tropospheric air temperature ($\delta \left( T \right)$., contours, K) and the three TEE terms (color shading K day^−1^) between the positive and negative phases of the KZI events along a latitudinal profile (across 70°E–80°E) over the western TP for 79-2016. Left column is for $\delta \left( {ADH} \right)$, the middle column is for $\delta \left( {HTAD} \right)$ and the right column is for $\delta \left( {DH} \right)$. **a**–**c** Are for summer (JJA), **d**–**f** are for autumn (SON), **g**–**i** are for winter (DJF), and **j**–**l** are for spring (MAM). The black dots denote significance of the TEE terms above the 0.05 level, after taking account of the efficient number of degrees of freedom (Zar 1984; Li et al. 2013). The solid, dashed and bold solid grey contour lines denote the positive, negative and zero values of air temperature difference. Black shaded area denotes the topography. The green triangle denotes the central Karakoram (36°N, 75°E)

There are significant changes in vertical motions associated with WTV variability over the western TP in all four seasons (Fig. 3). In summer (Fig. 3a, b) there are significant differences in vertical velocity between positive and negative KZI phases at multiple lower tropospheric levels including 500-hPa and 300-hPa. Significant downward (upward) anomalies are related to anti-cyclonic (cyclonic) WTV conditions, from the western slope (west of 70°E) of the TP at 500 hPa (Fig. 4a) and lower levels (not shown) up to and over the main body of the western TP high mountain area and the Tarim Basin at higher levels, which can also be observed in the vertical profile above the central western TP in Fig. 2a (contours). In the other three seasons (Fig. 3c–h), an anti-cyclonic (cyclonic) WTV causes anomalous downward (upward) vertical motions over the southwest slope of the TP that spreads across the main body of the western TP high mountain area, consistent with the vertical profile in Fig. 2b–d. We also note that in autumn, winter and spring, the differences in vertical velocity over the southwest slope of the TP are stronger than for other areas of the TP. This suggests that the anomalous downward (upward) vertical motions associated with the anti-cyclonic (cyclonic) WTV could be amplified by the topographic lifting effect of the west slope of the TP in summer and the lifting effect of the southwest slope of the TP in the other three seasons, but further numerical experiments are needed to verify this theory.

Considering that the Subtropical Westerly Jet is prevailing over the TP topography, its seasonal moments match well with the seasonal differences of the topographic lifting effects associated with WTV variability. As shown in Figure S1 (see also Fig. 1 of Li et al. 2018), in summer the axis of the Subtropical Westerly Jet moves to its most northern position (Figs. S1a and S2a), when the westerly blows across the west slope of the TP and produces lifting motions there in summer. In the other three seasons (Fig. S1 bcd and Fig. S2 bcd), the axis of the Subtropical Westerly Jet is located to the south of the TP topography, with strong westerly winds blowing across the southwest slope of the TP topography, and causing strong lifting motions at the southwest slope of the TP in autumn, winter and spring. As WTV variability has been demonstrated (Li et al. 2018) to be highly associated with the variability of the Subtropical Westerly Jet, the seasonal changes of the Jet could explain the seasonal differences of the vertical velocities and the topography lifting effects under WTV variability, but this needs further verification and is out of scope of this study.

Another interesting phenomenon is a horizontal “dipole-like” structure of sinking (rising) motion over the western TP, in contrast to the rising (sinking) motion over the southwest slope under an anti-cyclonic (cyclonic) WTV in both summer and spring. As evidenced in Fig. 3a, b, g, h, positive $\delta \left( w \right)$ is observed over the Central Himalaya (far Northern India, western Nepal), although it is not significant at the 0.05 level, which is opposite to the significant negative $\delta \left( w \right)$ over the central/far western TP. How the dipole-like vertical motion structure is generated and its impacts on WTV and TP climate variability are important questions that need further investigation, but are out of scope in this paper. In addition, the vertical air temperature shows an obvious northward tilting structure in Fig. 2, which is in particular remarkable in summer (Fig. 2a) and autumn (Fig. 2b). Why this northward tilting structure occurs is still an open question.

The existence of anomalous sinki (rising) motions in the middle of anti-cyclones (cyclones) is a common atmospheric phenomenon (Randall 2005). Additionally, the west slope and the southwest slope of the TP amplifies the vertical motions in the core volume of the WTV through mechanical lifting, which deserves more attention. More importantly, we find the area with vertical motions related to WTV variability at the near surface of the TP (500-hPa level) generally matches the area where the WTV impacts on $T_{2m}$ (Fig. 3b, d, f, h VS Fig. 2a–d). Similarly, in vertical profiles (Fig. 2), the column with higher rising motions generally overlaps the column with greatest temperature increase in all four seasons. This implies that adiabatic heating processes related to WTV variability could be the major control of mid-lower tropospheric and near-surface air temperatures over the western TP, supporting FL1718. However, since the centres of greatest temperature change are not always well matched with those of the greatest vertical velocity centres, especially in winter (Fig. 2c) and spring (Fig. 2d), further quantification of the contributions of adiabatic heating and other thermodynamic terms are still needed.

## Dominant adiabatic rising-expandg-cooling/sinking-compressing-warming processes in the mid-lower troposphere over the western TP

We composite the three major thermodynamic terms of the TEE (Holton 2004; see Eq. 8) by positive or negative KZI. The difference in the composited terms ($\delta \left( {ADH} \right)$, $\delta \left( {HTAD} \right)$ or $\delta \left( {DH} \right)$) for positive KZI events minus those for negative KZI events (see Sect. 2.3 and Eqs. 9 and 10) are then used to quantify their relative contributions to mid-lower tropospheric and near-surface temperature increases over the western TP. According to the thermal equilibrium law, the composited differences of the terms are also under equilibrium (see Eq. 11 in Sect. 2.3 for more details). By analysing the area-averaged $\delta \left( {ADH} \right)$,$\delta \left( {HTAD} \right)$ and $\delta \left( {DH} \right)$, as well as the zonal-averaged vertical profile of the three difference terms, over the central western TP, we demonstrate that adiabatic heating is the dominant process through which the WTV modulates tropospheric air temperature over the western TP high mountain area in all seasons.

### The area-averaged contributions of tropospheric thermodynamic processes over the western TP

We calculated the spatially averaged $\delta \left( {ADH} \right)$, $\delta \left( {HTAD} \right)$ and $\delta \left( {DH} \right)$ over the western TP high mountain area (above 1500 m and within 24.75°N–44.5°N, 60°E–90°E) at 500-hPa and 300-hPa levels for all four seasons. 500 hPa and 300 hPa levels are respectively the near-surface of the western TP high mountain area and the level with greatest air temperature changes in the mid-lower troposphere above the western TP. At both levels, the adiabatic rising-expanding-cooling/sinking-compressing-warming process is demonstrated to be the dominant thermodynamic process through which the WTV modulates temperature changes over the western TP high mountain area in all seasons.

Shown as Table 2, we see that therence in adiabatic heating, $\delta \left( {ADH} \right)$, is the largest positive term (among the three terms) that balances the sum of the other two terms for each season at both the 300-hPa and 500-hPa levels. For exale, for winter, $\delta \left( {ADH} \right)$ is the strongest among the four seasons, reaching 0.47 and 0.27 K day^−1^ at 300-hPa and 500-hPa respectively, which also coincides with the greatest vertical velocity difference, $\delta \left( w \right)$, over the western TP high mountain area (Fig. 3). In summer, in contrast, the mean difference in $\delta \left( {ADH} \right)$ is the weakest, reaching only 0.060 and 0.0365 K Day^−1^ at 300-hPa and 500-hPa respectively, which coincides with the weakest $\delta \left( w \right)$ over the western TP high mountain area (Fig. 2). However, this adiabatic heating difference is still a much bigger difference than for either of the other two thermodynamic terms in summer. As the air temperature increases in the positive relative to the negative KZI events (Fig. 2; also see Fig. 3 in Li et al. 2018), the term with the biggest positive difference is thereby the major thermodynamic process that causes the temperature increase in positive relative to negative KZI events (also see Sect. 2.3). Therefore, adiabatic heating (or vertical temperature advection) is the dominant process contributing to temperature changes over the wester TP high maintain area associated with WTV variability; adiabatic sinking (rising) causes temperature incases (decreases) in the mid-lower troposphere and at the near-surface under an anti-cyclonic (cyclonic) WTV. This theoretical analysis verifies the observational results shown in FL1718.

It should be noted here that the other two thermodynamic processes, horizontal temperature advection and diabatic heating, are also important contributors to temperature changes over the western TP high mountain area. For example, $\delta \left( {HTAD} \right)$ at the 500-hPa level is respectively 0.017 and 0.003 K day^−1^ in autumn and spring, suggesting that horizontal temperature advection at the near-surface of the western TP is important and positively contributes to temperature changes related to WTV variability, although its contribution is much smaller ($< 7^{ - 1}$ times) than that of adiabatic sinking. In addition, $\delta \left( {DH} \right)$ at 300-hPa in summer is 0.009 K day^−1^, which could be due to the latent heat release associated with vertical rising (Fig. 3a) and condensing air along the south slope of TP. It suggests that diabatic heating also positively contributes to the temperature increases related to an anti-cyclonic (cyclonic) WTV.

From above, we demonstrate that adiabatic heating is the dominant thermodynamic process modulating temperature changes associated with WTV variability over the western TP in all four seasons. We note that other thermal processes, horizontal temperature advection and diabatic heating, also have significant influence in some seasons, which assists the adiabatic heating to modulate or even dominate temperature changes in some sub-regions of the western TP under WTV variability, as the topography of the western TP is so rugged.

### Vertical distributions of zonal-averaged thermodynamic contributions over the central western TP

We also calculated the vertical profile of the zonal-averaged $\delta \left( {ADH} \right)$, $\delta \left( {HTAD} \right)$ and $\delta \left( {DH} \right)$ over the central western TP (between 70 and 80°E, as shown in Fig. 4), which demonstrates similar results to the previous section and provides more detailed proof.

In summer (Fig. 4a), $\delta \left( {ADH} \right)$ is a triple structure south-north across the western TP: under an anti-cyclonic (cyclonic) WTV, there is adiabatic cooling (heating) over the south slope of the western TP, adiabatic warming (cooling) over the central western TP highlands near the Karakoram, and then adiabatic cooling (heating) again over the north slope, the Kunlun Shan and the Tarim basin. The $\delta \left( {HTAD} \right)$ (Fig. 4b) essentially reverses the pattern of the $\delta \left( {ADH} \right)$. However, the $\delta \left( {DH} \right)$ (Fig. 4c) has essentially a ‘simple’ dipole with diabatic heating (cooling) over the south slope/Himalaya (along with the near-surface further north in the transect) but mid-tropospheric cooling (heating) above the Karakoram, the Kunlun Shan and the Tarim basin. In the other three seasons, both the $\delta \left( {ADH} \right)$ (Fig. 4d, g, j) and the $\delta \left( {HTAD} \right)$ (Fig. 4e, h, k) have a dipole structure that essentially balance each other, and the $\delta \left( {DH} \right)$ generally shows a weak contrast through the transect. There is substantial dominance of adiabatic heating (cooling) (Fig. 4d, g, j) over the south and centre of the transect (including the south slope and the gh mountain areas of the western TP) with substantial adiabatic cooling (heating) over the Tarim/far north under the anti-cyclonic (cyclonic) WTV. Therefore, across the south-north of the wider western TP area in the mid-to-lower troposphere, the $\delta \left( {ADH} \right)$ is generally balanced by the $\delta \left( {HTAD} \right)$, and e $\delta \left( {ADH} \right)$ is relatively small except for during the summer season. In addition, comparing the distribution of the $\delta \left( {ADH} \right)$ with the $\delta \left( w \right)$ (Fig. 4a, d, g, j VS Fig. 2a–d), we observe their similarity, which is reasonable as the adiabatic cooling/heating process is mainly caused by the vertical motions.

**Fig. 5:**
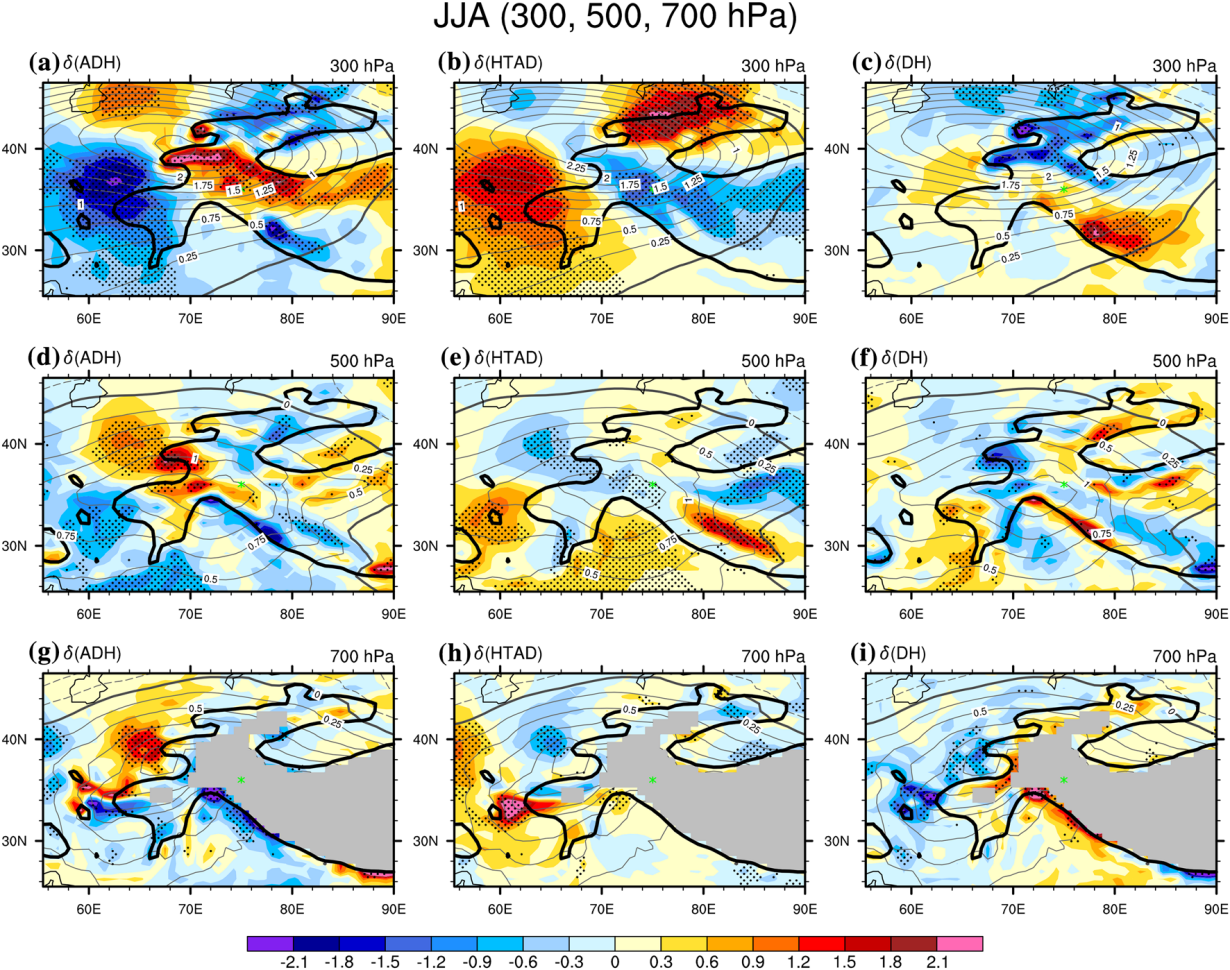
The difference of the tropospher air temperature ($\delta \left( T \right)$, contours, K) and the three TEE terms (color shading, K day^−1^) between the positive and negative phases of the KZI events on multiple levels over the western TP in summer (JJA) in 1979016. Left column is for $\delta \left( {ADH} \right)$, the middle column is for $\delta \left( {HTAD} \right)$ and the right column is for $\delta \left( {DH} \right)$. **a**–**c** Are for 300 hPa, **d**–**f** are for 500 hPa, **g**–**i** are for 850 hPa. The solid, dashed and bold solid grey contour lines denote the positive, negative and zero values of air temperature difference. The black dots denote significance of the TEE terms above the 0.05 level, after taking account of the efficient number of degrees of freedom (Zar 1984; Li et al. 2013). Bold-black-outlie denotes topography above 1500 m. Grey shaded area denotes the topography above the isobaric surface. The green star denotes the central Karakoram (36°N, 75°E)

If we compare the distributions of the $\delta \left( {ADH} \right)$ and that of the $\delta \left( T \right)$ (grey contours in Fig. 4), we can again verify the dominant of the adiabatic sinking (rising) process in driving the positive (negative) temperature anomaly the mid-to-lower troposphere over the western TP high mountain area under an anti-cyclonic (cyclonic) WTV. Shown as contours in Fig. 4a–c, the summertime $\delta \left( T \right)$ is generally positive over the wider TP area in the mid-to-lower troposphere, except for negative $\delta \left( T \right)$ observed below 700 hPa south of the TP, with the most positive $\delta \left( T \right)$ centred at 250 hPa in the mid-lower troposphere in summer. As the distribution of summertime $\delta \left( {ADH} \right)$ (color-shading in Fig. 4a) exhibits as a triple structure—“negative–positive–negative” values from the south to the north of the western TP area—so the contributions of the adiabatic rising-expanding-cooling/sinking-compressing-warming process to the temperature increases located above the western TP are opposite for contributions to temperature increases located to either the north or the south of the western TP. For areas right above the central westernP high mountain area, we observe significant positive $\delta \left( {ADH} \right)$ (color-shading in Fig. 4a), denotinthe positive contributions of the $ADH$ term to temperature increases (decreases) under anti-cyclonic (cyclonic) WTV (analysis methods based on Sect. 2.3). What’s more the centre with greatest positive $\delta \left( {ADH} \right)$, significant at the 0.05 level, is located nearer to, but a little south of, the greatest positive $\delta \left( T \right)$ centre above the western TP, demonstrating that summertime air temperature increases (decreases) at 250 hPa in the mid-lower troposphere right above the central western TP high mountain area are mainly contributed by adiabatic heating (cooling). In contrast, temperature increases in the mid-to-lower troposphere to the north and south of the western TP are mainly caused by the increase in horizontal temperature advection, as evidenced by the significant positive $\delta \left( {HTAD} \right)$ located there (Fig. 4b). In addition, we can also observe significant positive $\delta \left( {DH} \right)$ at the south slope and the near-surface of the western TP, denoting that diabatic heating (sensible heating, radiative heating, latent heating etc.) is also important in causing temperature increase to the west and for the southwest slope of the western TP (also see Fig. 5 for more details). However, above the central western TP high mountain area in summer, adiabatic sinking (rising) processes are generally the dominant driver of temperature variations in the mid-to-lower troposphere.

For autumn, winter and spring, the matching between the greatest positive $\delta \left( {ADH} \right)$ centres and the greatest positive $\delta \left( T \right)$ centres in mid-lower troposphere is much better than in summer, as evidenced by the two centre overlaps at 500 hPa–200 hPa within 30°E–40°E (Fig. 4d, g, j VS Fig. 4a). This demonstrates the dominant contribution of adiabatic heating (cooling) to the temperature increase (decrease) centering on the mid-lower troposphere over the western TP and neighbouring areas to its south under anti-cyclonic (cyclonic) WTV. The composited differences of the other two terms, $\delta \left( {HTAD} \right)$ and $\delta \left( {DH} \right)$, are negative near to the positive $\delta \left( T \right)$ centres in 500 hPa–200 hPa within 30°E–40°E, and are balanced by the adbatic term. Especially in autumn, the distribution of $\delta \left( T \right)$ is also a dipole structure (Fig. 4d), highly matching the distribution of the $\delta \left( {HTAD} \right)$ (Fig. 4e); both are positive over the south slope of the western TP and over the central western TP highlands near the Karakoram, but with negative values over the north slope, the Kunlun Shan and the Tarim basin. It means the diabatic heating process dominates the air temperature changes in the mid-to-lower troposphere over nearly the entire wider western TP area. For winter and spring, $\delta \left( T \right)$ is still positive over the Kunlun Shan and the Tarim basin, and mainly caused by the positive y $\delta \left( {HTAD} \right)$ (Fig. 4h, k). Therefore, the diabatic heating due to the vertical sinking (rising) motions is the dominant mechanism by which the WTV drives the temperature increase (decrease) centering at the mid-lower troposphere in all four seasons.

As mentioned above, we emphasize that horizontal temperature advection and diabatic heating are also important in summer in the mid-troposphere and at the near-surface over the western TP, although adiabatic rising-expanding-cooling/sinking-compressing-warming is the dominant mechanism. Shown as Fig. 4b, the $\delta \left( {HTAD} \right)$ is also positive and is overlapped with the center of temperature increase in the mid-lower troposphere, significant at the 0.05 level; suggesting the horizontal temperature advection also positively contributes to temperature changes associated with WTV variability. However, at the near-surface of the central western TP high mountain area, the y $\delta \left( {HTAD} \right)$ is negative, significant at the 0.05 level, suggesting it not positively contributing to the near surface air temperature increase. We also note that the summertime diabatic heating term is important at the near-surface of the central western TP high mountain area, as the $\delta \left( {DH} \right)$ (Fig. 4c) is positive, significant at the 0.05 level. Therefore, we conclude that horizontal temperature advection and diabatic heating are important in assisting the dominant effects of adiabatic heating in modulating the mid-lower tropospheric air temperature changes.

## Regional contributions of tropospheric thermodynamic processes

In this section, we further explore the regional contributions of different thermodynamic processes on multiple levels in the mid-lower troposphere in all four seasons, as a comparison to its vertical profile and the general average over the weern TP presented in the previous section. We again focus on three levels: 700 hPa, 500-hpa and 300-hPa, in the following analysis. We study the wider western TP area, denoted as the area within 25°–47°N, 55°–90°E. From these analyses, we provide more detailed features on how the adiabatic rising-expanding-cooling/sinking-compressing-warming process and other processes have effects on multiple levels over the western TP.

### Summer

For multiple levels above the wider western TP (Fig. 5), the magnitudes of the $\delta \left( {ADH} \right)$, $\delta \left( {HTAD} \right)$ and $\delta \left( {DH} \right)$ at 300 hPa (Fig. 5a–c) are generally stronger than that for lower levels (Fig. 5d–i), which coincides with a stronger $\delta \left( T \right)$ at 300 hPa than at lower levels. This is consistent with the vertical profiles shown in the previous section which reported that the temperature changes and the TEE terms’ contributions in the mid-to-lower troposphere are centred at 250–400 hPa in summer.

We also find the distribution the $\delta \left( {ADH} \right)$ exhibits as a triple structure, which is most pronounced at 300 hPa (Fig. 5a) and is weaker at lower levels (Fig. 5d, g), matching the basic features of the vertical profiles in the previous section. Shown as Fig. 5a, under anti-cyclonic (cyclonic) WTV, there is adiabatic cooling (heating) over either the southwest or the northeast of the wider western TP area, but adiabatic heating (cooling) over the middle of the wider western TP, including the highlands centring on the Karakoram and the northern TP and even the plains area near the Caspian Sea. The distribution of $\delta \left( {HTAD} \right)$ (Fig. 5b) essentially shows the reverse of this pattern. The $\delta \left( {DH} \right)$ (Fig. 5c) exhibits an essentially dipole distribution; with diabatic heating (cooling) over the north part of the western TP, and with diabatic cooling (heating) over the south part of the western TP centred on the south slope. So the distributions of the three TEE terms contributions are generally similar to those for the vertical profile reported in the previous section.

Comparing the $\delta \left( T \right)$ with the $\delta \left( {ADH} \right)$, we find that significant positive $\delta \left( {ADH} \right)$ is always located near to centres with the greest $\delta \left( T \right) {\text{at}}$ all three tropospheric levels in summer. Shown as contours in Fig. 5a, d, g, positive $\delta \left( T \right)$ are centred on the west tail of the TP at all three levels, indicating the air temperature surrounding and above the western TP in anti-cyclonic (cyclonic) WTV events is warmer (cooler) than that in cyclonic (anti-cyclonic) WTV events. The significant positive $\delta \left( {ADH} \right)$, as well as the significant negative $\delta \left( w \right)$ (as Fig. 2b, d in Sect. 3.2, but 700 hPa level is not shown), are also distributed over the west tail of the TP at all three levels (Fig. 5a, d, g), generally matching the highest positive $\delta \left( T \right)$ centre; this suggests the adiabatic sinking (rising) process is the biggest positive contributor to temperature increases (decreases) over the western TP high mountain area under anti-cyclonic (cyclonic) WTV. In contrast, an area with negative $\delta \left( {ADH} \right)$ (e.g. over the southwest and northeast flanks of the wider western TP) has smaller temperature increases or smaller $\delta \left( T \right)$, although the $\delta \left( {HTAD} \right)$ and the $\delta \left( {DH} \right)$ usually positively contributes to the temperature increases there. This suggests the horizontal temperature advection and diabatic heating process are generally in more minor roles in driving the air temperature changes associated with WTV variability. Therefore, adiabatic rising-expanding-cooling/sinking-compressing-warming is the major driving mechanism in determining the thermodynamic structures or temperature changes in the mid-to-lower troposphere under WTV variability.

Although the adiabatic rising-expanding-cooling/sinking-compressing-warming is the dominant mechanism, summertime diabatic heating is still important, consistent with the results based on the vertical profile. Actually, the stronger diabatic heating effect is one of the biggest differences between summer and other seasons. Firstly, for the southern slope of the western TP, the positive contributions of diabatic heating are observed from the slope of the western TP at 700 hPa to above it in the mid-high troposphere centred at 300 hPa. As evidenced in Fig. 5c, f, i, we find positive $\delta \left( {DH} \right)$ over the south edges of the western TP at all three levels, centred on the south slope of the TP near 70°–85°E, significant at the 0.05 level, demonstrating the diabatic heating processes are positively contributing to the air temperature increases (decreases) in the troposphere under the anti-cyclonic (cyclonic) WTV. The increased (decreased) adiabatic heating over the south slope under anti-cyclonic (cyclonic) WTV could be due to related to enhanced (weakened) sensible, radiative, or latent heating. There are also ascending motions (Fig. 3a–d) and significant increases of the total cloud cover (TCC) (Fig. S4a) occurring over the south slope in the anti-cyclonic WTV relative to the cyclonic WTV, i.e. $\delta \left( w \right) > 0$ (Fig. 3a–d) and $\delta \left( {TCC} \right) > 0$ (Fig. S4a). Given that more cloudiness is associated with enhanced condensation and more latent heat release, but blocks more input of shortwave radiation, the enhanced diabatic heating over the south slope under anti-cyclonic WTV could be mainly induced by more latent heat release from the enhanced condensation process associated with ascending motions, and vice versa. This is consistent with the studies of Yu (2009) and Yu et al. (2011a, b), which documented that latent heat release is abundant on the south slope of the TP associated with vertical motions in summer. Secondly, over the west edge or the west tail of the western TP, the positive contribution to air temperature changes in lower levels fr the diabatic heating can be verified by the significant positive $\delta \left( {DH} \right)$ at the 700 hPa level over the west slopes of the western TP. As this significant positive $\delta \left( {DH} \right)$ is near to the center of the WTV, which has significant descending motions, i.e. $\delta \left( w \right) < 0$ (Fig. 2a–d), and the TCC is significantly decreased over the slopes of the west tail of the TP in the anti-cyclonic WTV relative to the cyclonic WTV, i.e. $\delta \left( {TCC} \right) < 0$ (Figures not shown), it suggests the increase (decrease) in input of shortwave radiation due to the decrease (increase) of TCC could be the major reason for significant diabatic heating (cooling) effects at the west tail of the TP under anti-cyclonic (cyclonic) WTV. Therefore, the effects of latent heat release over the south slope of the western TP, and cloud-radiation effects at low levels over the west slope of the western TP are two important diabatic processes which enhance the impacts of the WTV on air temperature. Detailed quantification of the diabatic heating processes in summer deserves further examination but is out of the scope of the current study.

At the edge of the western TP high mountain area, there are three regions where enhanced warm horizontal temperature advection significantly contributes to temperature increase, although these contributions are not near to the centres of air temperature changes under WTV variability. Two significant anomalous warm advection centres are located in the northwest and west of the western TP at both the 500-hPa and 300-hPa levels (Fig. 5b, e), which even penetrate down and show similar signals at the 700 hPa level (Fig. 5h), and are caused by the anomalous anti-cyclonic WTV (see Fig. 2a) which brings thermal energy from the warm south to the cold north. Another anomalous warm advection center is located at the southwest slope of the TP near to 75°–90°E at the 500-hPa level (Fig. 5e) only, which is mainly due to the anomalous east winds related to the anti-cyclonic WTV bringing thermal energy from the warm southern TP to the cold southwest TP (Figure not shown) at the near-surface of the TP. Moreover, comparing Figs. 5b, e, f and 2a, we can see all the above three mentioned regions with significant positive $\delta \left( {ADH} \right)$ centers are located along horizontal anti-cyclonic wind vectors (Fig. 2a) associated with the WTV, circling around the central core column of the WTV. Therefore, horizontal temperature advection also plays an important role in causing temperature increase at the edge of the western TP.

As the WTV is coupled (Forsythe et al. 2017) with the South Asia summer monsoon (SASM), the horizontal temperature advection associated with the warm monsoonal circulations could also play a role in modulating the temperature changes. After checking, we do find the horizontal temperature advection term positively contributes the temperature increase over the SASM’s prevailing area (Li and Zeng 2002, 2003), i.e. northern Pakistan and northern India, as evidenced by the significant positive $\delta \left( {HTAD} \right)$ located near to the centre of greatest temperature increases at the 700 and 500 hPa levels (Fig. 5e, f). However, these significant positive $\delta \left( {HTAD} \right)$ are confined to the plain areas over northern Pakistan and northern India and some parts of the adjacent lower slopes of the western TP. They do not however extend into the central western TP high mountain area, apart from on the southwest slope of the TP near to 75°–90°E at the 500-hPa level, where the horizontal advection is significantly contribution to the temperature increase. This suggests a limited impact of warm or cold air currents originating from monsoonal areas over the western TP high mountain areas relative to influence on temperature variability in the plains. The other possible way in which the SASM influences the WTV and air temperature over the TP could lie in the diabatic heating over the south slope of the TP, which could be induced by SASM circulations on lower levels through topographical lifting effects. Whether the significant horizontal temperature advection and diabatic heating over the southwest slope of the TP originates from the monsoonal circulation needs further investigation but is beyond the scope of the current study.

In previous studies (FL1718), the adiabatic heating is emphasized as the major mechanism by which the WTV impacts air temperature in summer. Here, we have verified that adiabatic heating and cooling effects due to rising/expanding and sinking/compressing are the dominant thermodynamic process through which the WTV influences the tropospheric temperature over the central western TP high mountain area. Moreover, we find that the other two thermodynamic processes, horizontal temperature advection and diabatic heating, are also important drivers of tropospheric temperature variability at the edges of the western TP. In particular, diabatic heating related to latent heat release and cloud-radiation interactions at the west and south edges of the western TP are important in enhancing or compensating the driving effects of diabatic heating.

### Autumn, winter and spring

**Fig. 6:**
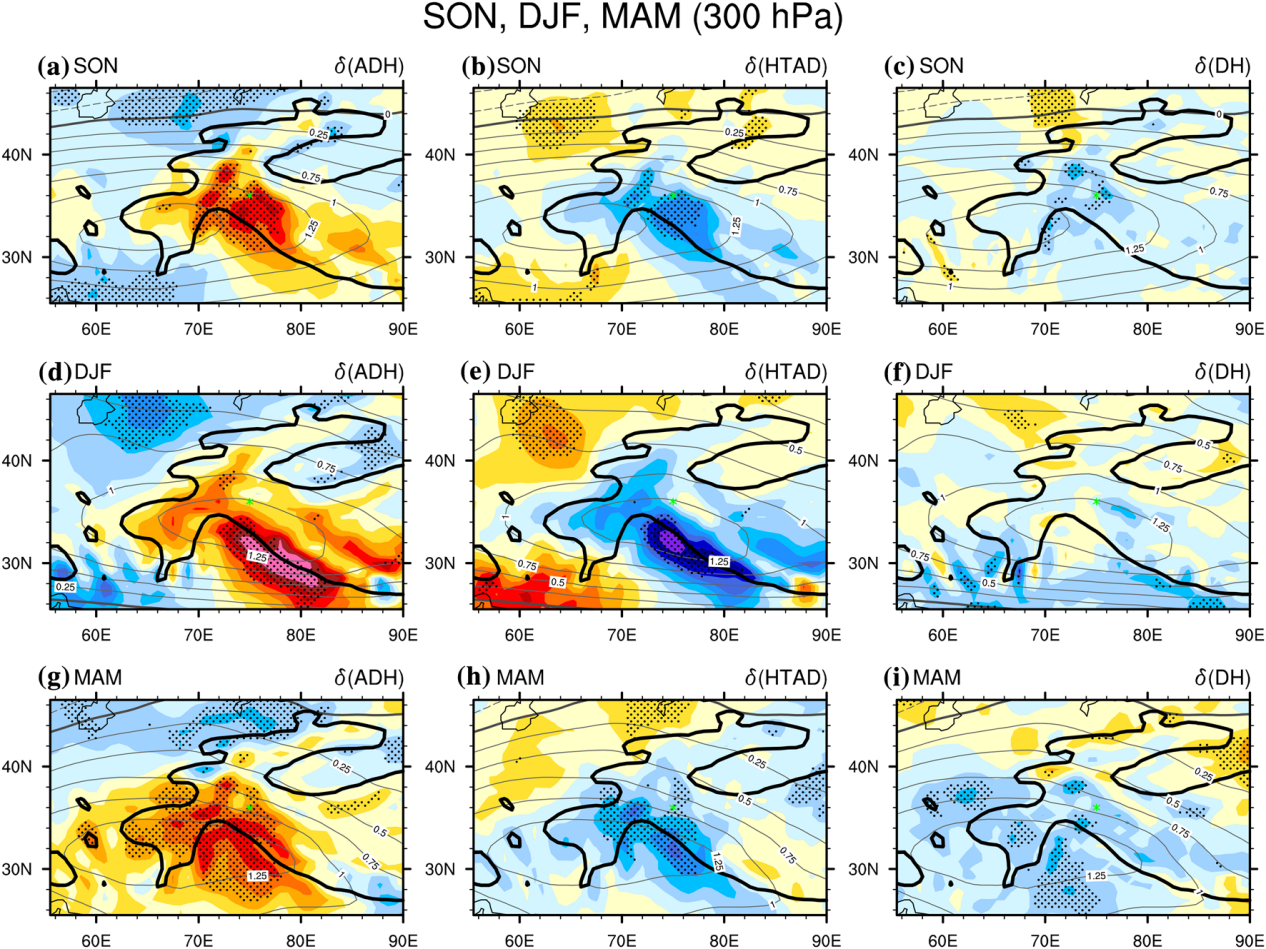
The difference of the tropospheric air temperature ($\delta \left( T \right)$, contours, K) and the three TEE terms (color shading K day^−1^) between the positive and negative phases of the KZI events at 300 hPa over the western TP in 1979–2016. Left column is for $\delta \left( {ADH} \right)$, the middle column is for $\delta \left( {HTAD} \right)$ and the right column is for $\delta \left( {DH} \right)$. **a**–**c** Are for autumn (SON), **d**–**f** are for winter (DJF), and **g**–**i** are for spring (MAM). The black dots denote significance of the TEE terms above the 0.05 level, after taking account of the efficient number of degrees of freedom (Zar 1984; Li et al. 2013). The solid, dashed and bold solid grey contour lines denote the positive, negative and zero values of air temperature difference. Bold-black-outlie denotes the topography above 1500 m. The green triangle denotes the central Karakoram (36°N, 75°E)

Relative to summer, the thermodynac processes in other three seasons are much simpler, as the dominance of adiabatic rising-expanding-cooling/sinking-compressing-warming is quite uniform over the western TP. Shown as Fig. 6a, d, g, the positive $\delta \left( {ADH} \right)$ at the 300 hPa level is centred on the south slope of TP between 70°E and 80°E, significant at the 0.05 level, and the positive $\delta \left( {ADH} \right)$ is spread to nearly the entire western TP. Meanwhile, either the $\delta \left( {HTAD} \right)$ (Fig. 6b, e, h) or the $\delta \left( {DH} \right)$ (Fig. 6c, f, i) at the 300 hPa level is generally negative over the entire western TP, the sum of which are balanced by the positive $\delta \left( {ADH} \right)$. Moreover, the patterns of the positive $\delta \left( T \right)$ (black solid contours in Fig. 6) matches well with that of the positive $\delta \left( {HTAD} \right)$., i.e. the highest $\delta \left( T \right)$ is always located near to the highest $\delta \left( {ADH} \right)$; in contrast, the area with negative $\delta \left( {ADH} \right)$ usually has a smaller or negative $\delta \left( T \right)$, although the $\delta \left( {HTAD} \right)$ or the $\delta \left( {DH} \right)$ could be positively contributing to the temperature increases there. It demonstrates that adiabatic sinking/compressing (rising/expanding) is the overwhelming driver causing air temperature increases (decreases) under the anti-cyclonic (cyclonic) WTV at the 300 hPa level in these seasons.

**Fig. 7:**
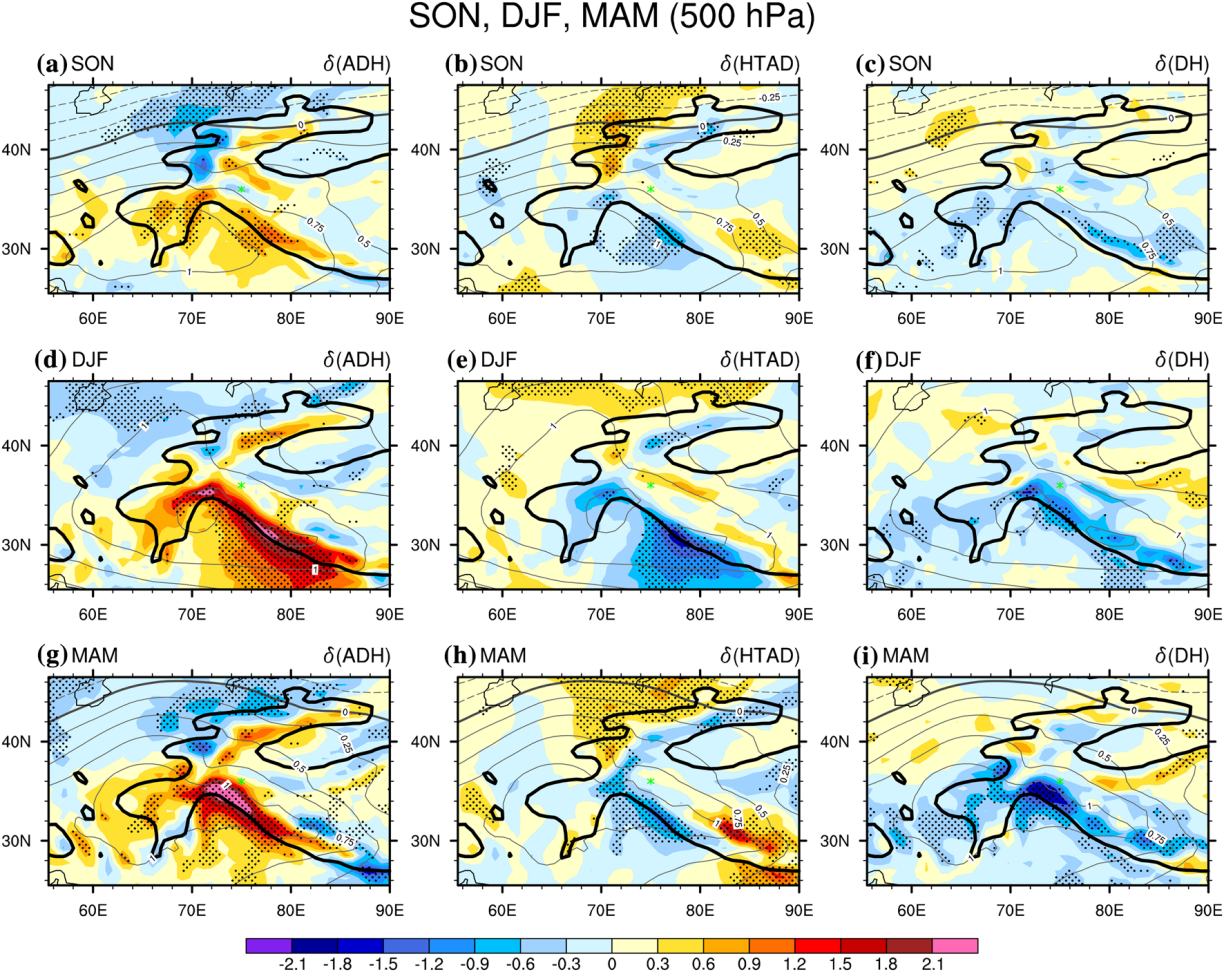
Same as Fig. 5, but at 500 hPa level

At 500 hPa (Fig. 7), the near-surface level of the western TP, the dominance of adiabatic rising-expanding-cooling/sinking-compressing-warming to temperature change is similar to that at the 300 hPa level (Fig. 6), except that the significant contribution from adiabatic rising-expanding-cooling/sinking-compressing-warming over the western TP high main area is (spatially) contracted around the south slope of the TP. Another difference is that horizontal temperature advection becomes more important at 500 hPa. For example, the $\delta \left( {HTAD} \right)$ is positive northwest and southeast of the western TP in both autumn (Fig. 7b) and spring (Fig. 7h), significant at the 0.05 level; the $\delta \left( {HTAD} \right)$ over the central western TP also becomes positive, but it is not significant at the 0.05 level. The contribution from diabatic heating also becomes more important over the northeast TP at 500 hPa in spring (Fig. 7f) and winter (Fig. 7i). However, all the positive contributions of the horizontal temperature advection are far away from the large temperature responses at 500 hPa in all three seasons, suggesting it has only a minor contribution to the temperature changes. In spring, over the slopes of the west tail of the TP (30°N–38°N, 62°E–70°E) at 700 hPa (Figure not shown), near the centre of the anti-cyclonic (cyclonic) WTV, $\delta \left( {AD} \right)$ is positive and significant at 0.05 level, suggesting that diabatic heating (cooling) at the surface of the slopes also positively contributes to temperature increases (decreases), enhancing the dominant influences of the adiabatic sinking/compressing (rising/expanding) process above it (Figure not shown). Therefore, adiabatic rising-expanding-cooling/sinking-compressing-warming is also the dominant thermodynamic process at the near-surface level over the western TP, but the other two thermodynamic processes are more important at the near-surface than at higher levels of the atmosphere.

Li et al. (2018) showed that the westerlies over the south slope of the TP in these three seasons are much stronger than during summer, as the Subtropical Westerly Jet is located south of the TP in these three seasons (see Figs. S1 and S2). This implies that the lifting effects of the south slope of the TP topography and the climatological distributions of the circulations could be very important to the behaviour of the adiabatic heating process. As a result, the temperature responses to WTV variability may be substantially enhanced in comparison with vortices over regions with simpler topography. It may be even that the location and structures of the WTV are induced by the interaction of the westerly jet with this topographic uplift. This is a very interesting line of inquiry which requires substantial further investigation but is nevertheless beyond our current research scope.

## Dominant role of stratospheric adiabatic rising-expanding-cooling/sinking-compressing-warming in modulating the stratospheric air temperature changes

The WTV not only influences air temperature in the troposphere but also impacts the air temperature in the stratosphere. During the positive (negative) KZI events, the WTV has an anti-cyclonic (cyclonic) wind structure, which causes temperature decreases (increases) in lower stratosphere, and the temperature increases (decreases) in the middle-to-lower troposphere, resulting in a vertical “dipole” structure between the middle-to-lower tropospheric and the lower stratospheric air temperature above the western TP (FL1718). The temperature decreases (increases) in the lower stratosphere between the positive and negative KZI events is verified well by the negative value of the averaged $\delta \left( T \right)$ at the 70 hPa level over the western TP, which ranges from − 0.99 to − 1.95 K among four seasons (Table 2). In this section, we further explore the contributions of various thermodynamic processes to the lower stratospheric air temperature changes under the context of the WTV variability.

To begin with, we find the adiabatic heating process is still the dominant driver of the temperature changes in lower stratosphere associated with WTV variability. It is demonstrated by the area-averaged value of the $\delta \left( {ADH} \right)$ over the western TP, i.e. above 1500 m and within 24.75°N–44.5°N, 60°E–90°E, at the 70 hPa level (shown as Table 2), which is the only negative term among the three terms in all four seasons. It means the temperature decreases (increases) in the lower stratosphere is positively contributed to by the adiabatic rising/expanding (sinking/compressing) process under the anti-cyclonic (cyclonic) WTV. This is consistent with previous studies (FL1718) which have documented the significant rising motions in the lower stratosphere over the western TP under positive WTV events relative to negative WTV events in all four seasons (Fig. 7).

**Fig. 8:**
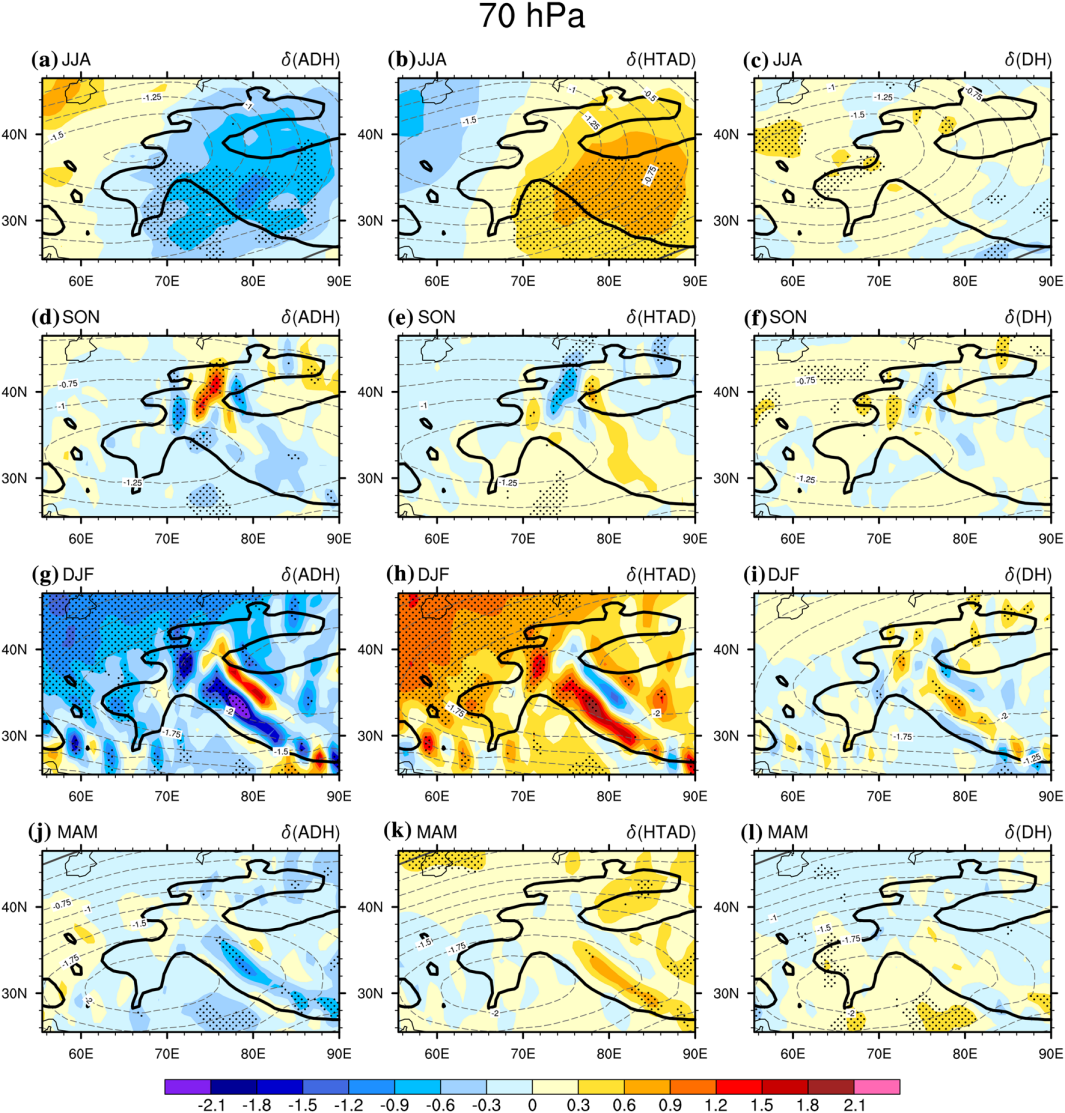
The difference of the stratospheric air temperature ($\delta \left( T \right)$, contours, K) and the three TEE terms (color shading, K day^−1^) between the positive and negative phases of the KZI at the 70 hPa level (the lower stratosphere) over the western TP in sum (JJA) in 1979–2016. Left column is for $\delta \left( {ADH} \right)$, the middle column is for $\delta \left( {HTAD} \right)$, the right columis for $\delta \left( {DH} \right) .$**a**–**c** Are for summer, **d**–**f** are for autumn, **g**–**i** are for winter, and **j**–**l** are for spring. The solid, dashed and bold solid grey contour lines denote the positive, negative and zero values of air temperature difference. The black dots denote significance of the TEE terms above the 0.05 level, after taking account of the efficient number of degrees of freedom (Zar 1984; Li et al. 2013). Bold-grey-outlie denotes topographybove 1500 m

**Fig. 9:**
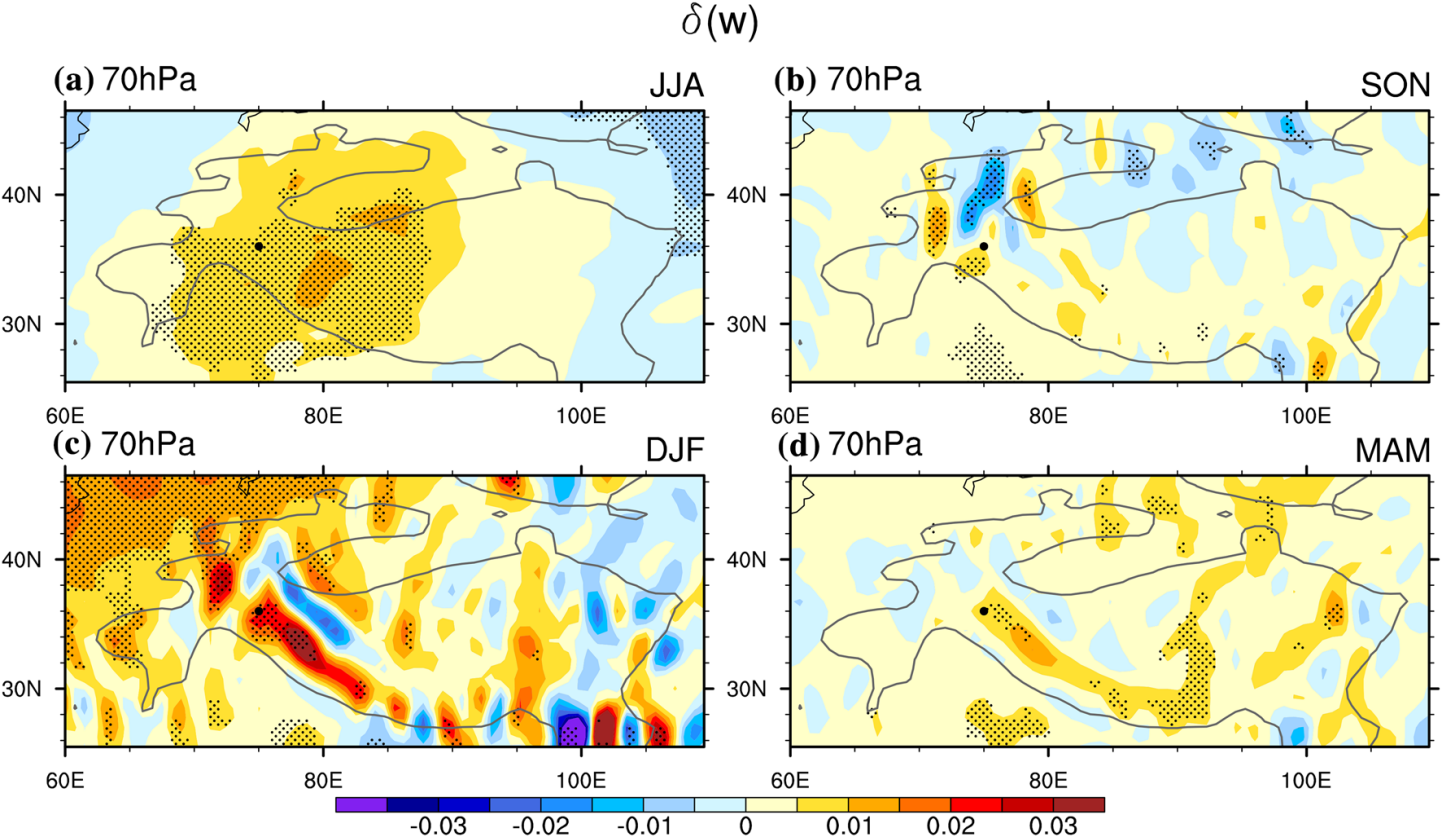
Composited differences of the vertical velocity ($\delta \left( w \right),$ color shading, 10^−1^ Pa s^−1^) between positive and negative KZI events (positive minus negative phase) at 70 hPa level above the Western Tibetan Plateau in **a** summer (JJA), **b** autumn (SON), **c** winter (DJF), and **d** spring (MAM) for 1979–2016. The significant correlations above the 0.05 level are stippled, after taking account of the efficient number of degrees of freedom (Zar 1984; Li et al. 2013). Bold black dot denotes the central position (36°N, 75°E) of the Karakoram focus area. Bold-grey-outline denotes topography above 1500 m

The determining role of the local adiabatic rising-expanding-cooling/sinking-compressing-warming processes on the temperature changes in the lower stratosphere can also be verified in the (planar) maps of the composited difference of the three major TEE terms (Fig. 8). In summer, the dominant role of the adiabatic rising-expanding-cooling/sinking-compressing-warming process is especially clear. Shown as the color shading in Fig. 8a, the $\delta \left( {ADH} \right)$ is negative over the western TP at 70 hPa level, significant at 0.05 level. As the negative $\delta \left( {ADH} \right)$ is coinciding with the distribution of the downward $\delta \left( w \right)$ (Fig. 9a), it suggests that the adiabatic rising/expanding process significantly contributes to cooling down the air temperatures in the lower stratosphere above the western TP, and vice versa. Given that the air temperature in the lower stratosphere is colder than normal for positive KZI events relative to negative KZI events, as evidenced by the negative $\delta \left( T \right)$ over the over the western TP, we conclude that the adiabatic rising-expanding (sinking-compressing) process positively contributes to the lower stratospheric temperature decreases (increases) associated with the anti-cyclonic (cyclonic) WTV. On the contrary, the other two thermodynamic processes negatively contribute to the lower stratospheric temperature changes, as both the $\delta \left( {HTAD} \right)$ and $\delta \left( {DH} \right)$ are positive over the western TP. Therefore, adiabatic rising-expanding-cooling/sinking-compressing-warming dominates the lower stratospheric temperature changes under WTV variability in summer.

Similarly, in other seasons (Fig. 8d, g, j) adiabatic rising-expanding-cooling/sinking-compressing-warming still dominates the lower stratospheric temperature changes (see significant negative values of the $\delta \left( {ADH} \right)$. over the south of the western TP). This distribution of the negative $\delta \left( {ADH} \right)$ coincides with the distributions of the upward vertical velocities in these seasons (see Fig. 9b–d). Furthermore, we also note that the uncertainty of the dominant thermodynamic processes in the lower stratosphere in these seasons is relatively bigger than in summer, with negative $\delta \left( {ADH} \right)$. showing less spatially uniform signals over the entire western TP. Especially, in autumn, significant positive $\delta \left( {ADH} \right)$ is observed over the north part of the western TP (Fig. 8d), suggesting adiabatic rising-expanding-cooling/sinking-compressing-warming processes are not dominating the thermal balance in this area. As $\delta \left( {HTAD} \right)$ and $\delta \left( {DH} \right)$ are negave in the north part of the western TP, significant at 0.05 level (Fig. 8e, f), this suggests the horizontal advection and diabatic heating positively contribute to temperature decreases (increases) under anti-cylconic (cyclonic) WTV in this area. In winter (Figure ghi), similarly adiabatic rising-expanding-cling/sinking-compressing-warming does not contribute positively in the north part of the western TP. Comparing the $\delta \left( {ADH} \right)$ of these three seasons in the lower stratosphere with that in the mid-lower troposphere (in previous section), we can also see the signals of $\delta \left( {ADH} \right)$ in the lower stratosphere are more uniform over the western TP than in the lower troposephere, suggesting the dominant driving role of the adiabatic rising-expanding-cooling/sinking-compressing-warming process has less uncertainty in the lower troposphere. Regardless, the adiabatic rising-expanding-cooling/sinking-compressing-warming process is still verified as the dominant driver in the lower stratosphere, although comprising relatively larger uncertainties than in the troposphere. Therefore, the vertical “dipole” structure between the middle-to-lower tropospheric and the lower stratospheric air temperature above the western TP is mainly caused by the adiabatic rising-expanding-cooling/sinking-compressing-warming process under WTV variability at both levels.

## A case study over the Karakoram area

The Karakoram area is located at the central area of the western TP, which has received considerable attention in previous studies (e.g., Hewitt 2005; Gardelle et al. 2012; Jacob et al. 2012; Kääb et al. 2012; Pratap et al. 2016; Bolch et al. 2017; Zhou et al. 2017). This is due to the fact that glaciers in the Karakoram have been relatively stable or even expanded in recent years—termed the “Karakoram anomaly”—compared to glaciers further east in the TP which have shown rapid melting. It is also the location with reference to which the WTV was identified (FL1718). It is therefore particularly interesting to study the contributions of thermodynamic processes to responses in air temperature over the Karakoram related to WTV variability. We respectively composite the three TEE terms over the Karakoram area according to the positive and negative phases of the KZI, using the same method as previously (see Sect. 2c). The Karakoram area is defined as a square area from 35 to 37°N and 74 to 76°E (denoted as green square in Fig. 1), around 400 km^2^ in area. The area averages of the composited difference of the TEE thermal terms between the positive and negative phases of KZI from the ground surface to the 50-hPa level, covering the mid-lower troposphere and lower stratosphere.

**Fig. 10:**
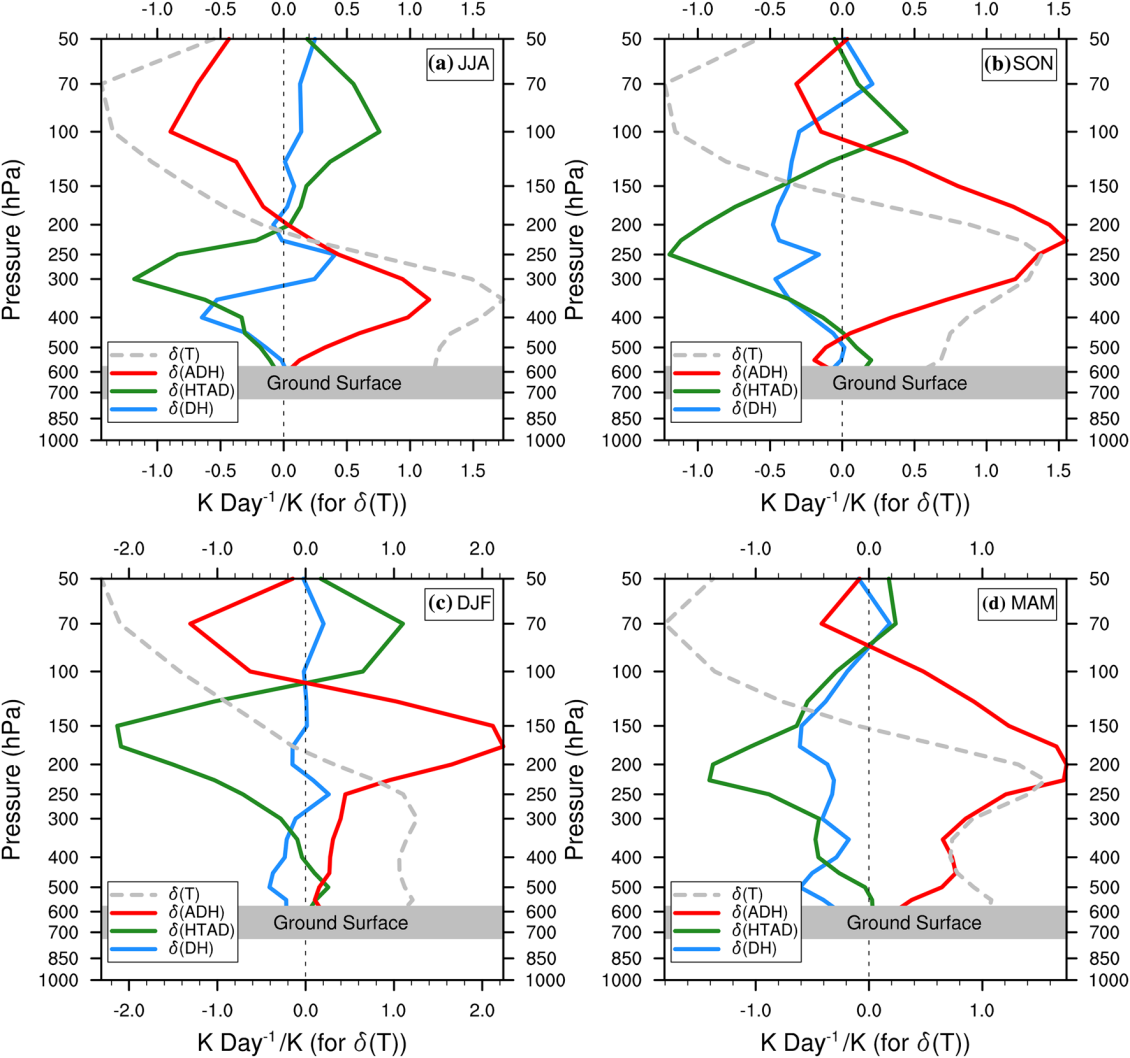
The averaged difference of the three composited three TEE terms (K Day^−1^) and the air temperature ($\delta \left( T \right)$, grey dash curve, K) between positive and negative phases of the KZI over the Karakoram area (35°N–37°N, 74°E–76°E) for **a** summer (JJA), **b** autumn (SON), **c** winter (DJF) and **d** spring (MAM) at multiple levels for 1979–2016. The red line is $\delta \left( {ADH} \right)$, the green line is $\delta \left( {HTAD} \right)$, the blue line is $\delta \left( {DH} \right)$. The horizontal grey-shading belt denotes ground surface of the Karakoram

As with the general feature over the western TP, the air temperature is warmer in the middle-to-lower troposphere and is colder in the lower stratosphere during positive KZI phases than in negative KZI phases above the Karakoram area, exhibiting as a “dipole” structure in the vertical direction. This is evidenced by the positive $\delta \left( T \right)$ (grey dashed line in Fig. 10) below 150–200 hPa, and the negative $\delta \left( T \right)$ above it. In addition, the $\delta \left( T \right)$ in the lower stratosphere shows the maximum cooling at 70 hPa, which verifies choosing 70 hPa as a representative level for analysing the thermodynamic processes in the lower stratosphere.

The dominant role of the adiabatic rising-expanding-cooling/sinking-compressing-warming process can be demonstrated well by the matching between the area averaged $\delta \left( T \right)$ and the $\delta \left( {ADH} \right)$: comparing the averaged $\delta \left( T \right)$ (grey dashed lines in Fig. 10) and the $\delta \left( {ADH} \right)$ (red solid lines in Fig. 10) above the Karakoram in Spring, Summer and Autumn. Winter however shows noisy results as the footprint for spatial aggregation happens to be crossed by a boundary between rising and sinking air (Fig. 2) for composited differences in that season. This is the source of the (sine) ‘waveform cancelling’ appearance over the Karakoram case study area in winter. The negative $\delta \left( T \right)$ in the lower stratosphere is centered at 70 hPa where the $\delta \left( {ADH} \right)$ is also negative in all seasons. This suggests that the air temperature decrease (increase) in the lower stratosphere under the anti-cyclonic WTV is driven by the cooling effects of the adiabatic ring/expanding (sinking/compressing) process. Similarly, a positive $\delta \left( T \right)$ is observed below 100–150 hPa (the transition layers between the troposphere and stratosphere) in the mid-lower stratosphere where the $\delta \left( {ADH} \right)$ is also positive, suggesting the air temperature increases (decreases) in the lower troposphere are driven by heating (cooling) effects of the adiabatic sinking/compressing (rising/expanding) process.

Although the adiabatic rising-expanding-cooling/sinking-compressing-warming is generally the dominant thermodynamic process driving the air temperature changes in both the lower stratosphere and the mid-lower troposphere, there are exceptions at the very near-surface in autumn and winter as mentioned above. In autumn (Fig. 9b) at the near-surface below 450-hPa over the Karakoram, the contributions of the horizontal temperature advection becomes dominant ($\delta \left( {HTAD} \right) > 0$), as the only term positively contributing to the energy balance, and the contribution from adiabatic sinking becomes negative ($\delta \left( {ADH} \right) < 0$). This is coincident with the non-significant impact of the WTV on $T_{2m}$ over the Karakoram in autumn (as shown as Fig. 2b). This implies that adiabatic sinking is the major way through which the WTV conducts its influence on near-surface air temperatures over the western TP: the lack of adiabatic rising-expanding-cooling/sinking-compressing-warming processes over the Karakoram in autumn blocks the impact of the WTV on near-surface air temperature in this region. In winter (Fig. 10c), the contribution from horizontal temperature advection becomes positive ($\delta \left( {HTAD} \right) > 0$) below 400-hPa, and even higher than that from adiabatic heating ($\delta \left( {HTAD} \right) > \delta \left( {ADH} \right)$ and $\delta \left( {ADH} \right) > 0$) at around 500-hPa, suggesting the contribution from both adiabatic heating and horizontal temperature advection are both important for temperature increases over the Karakoram associated with WTV variability in winter.

It should be emphasized that in summer and spring we find that adiabatic sinking (rising) is the dominant process—when comparing the composited positive and negative WTV phases—throughout the middle-to-lower troposphere (between the ground surface and the 300-hPa level) determining the temperature increases (decreases) associated with the WTV variability over the Karakoram. As evidenced in Fig. 10a, d, the mean vertical advection of potential temperature is the biggest positive contributor: the $\delta \left( {ADH} \right)$ reaches 0.33 K Day^−1^ and 0.64 K Day^−1^ at 500-hPa in summer and spring, respectively, which balances the contributions from the other two terms over the Karakoram in the same season. This means that the increase (decrease) of air temperature at the near-surface and in the mid-lower troposphere over the Karakoram associated with positive (negative) phases of the KZI in summer and spring is mainly conducted by increases (decreases) of vertical advection (red line), which offsets the negative contribution from horizontal advection (green line) and diabatic heating (blue line). This verifies the results from Forsythe et al. (2017) which suggested adiabatic processes were the dominant processes responding to summertime $T_{2m}$ changes under WTV variability over the Karakoram.

## Discussion and conclusions

By diagnosing the major terms of the thermal energy equations using ERA-Interim data, we have quantified the relative contributions of each thermodynamic term to air temperature changes in both the mid-lower troposphere and the lower stratosphere over the western TP under WTV variability. As the $T_{2m}$ changes is generally a present a profile cut out from the air temperature changes on multiple isobaric levels in the lower troposphere, but is either decayed or enhanced by some surface effects of the western TP topography. As a result, the thermodynamic processes at the near-surface level over the western TP at around 500 hPa can generally explain the $T_{2m}$ responses over the western TP high mountain areas. In summer and spring, however, we find that diabatic heating at near-surface levels below 500 hPa is also important above the western TP. We also examined the Karakoram region (35°N–37° N, 74°E–76°E) as a case-study, as temperature changes in the Karakoram are highly interesting to glaciologists who study the “Karakoram anomaly” (e.g., Hewitt 2005; Gardelle et al. 2012; Jacob et al. 2012; Kääb et al. 2012; Pratap et al. 2016; Bolch et al. 2017; Zhou et al. 2017; Forsythe et al. 2017).

In conclusion:

1. We find the climatological mean absolute horizontal wind speed over the near surface of the central western TP high mountain area is much smaller than its surrounding areas, which confines the influences of horizontal temperature advection on $T_{2m}$ over the central western TP. We also find significant sinking (rising) motions throughout the mid-low troposphere centered on the central western TP in summer and the south slope of the TP in other seasons during the anti-cyclonic (cyclonic) WTV.
2. As the area-averaged over western TP high mountain area is the biggest positive term in the troposphere, we demonstrate that adiabatic rising-expanding-cooling/sinking-compressing-warming is the dominant process through which the WTV modulates the tropospheric air temperature over the western TP in all seasons. The vertical profile of the zonal-averaged above the central western TP generally demonstrates the same results.
3. We find the thermodynamic processes in summer are relatively more complicated than in other seasons, as adiabatic rising-expanding-cooling/sinking-compressing-warming dominates the middle-to-lower tropospheric temperature responses in the central western TP high mountain areas, while horizontal temperature advection and diabatic heating plays an important role along the margins of the western TP. In other seasons, the dominance of the adiabatic heating on tropospheric temperature changes is relatively uniform over the entire western TP, centered on the south slope of the TP.
4. We find that adiabatic rising-expanding-cooling/sinking-compressing-warming also dominates lower stratospheric temperature responses to the KTV over the western TP, although there are relatively larger uncertainties existing for the stratosphere than for the troposphere.
5. We demonstrate that adiabatic heating is the controlling factor for $T_{2m}$. changes over the Karakoram in summer and spring. We further demonstrate that both adiabatic heating and horizontal temperature advection are important for $T_{2m}$ changes over the Karakoram in winter. However, we find that horizontal temperature advection is the dominant thermodynamic process related to WTV variability at the near-surface of the western TP (below 450-hPa) over the Karakoram in autumn; as a result, the impact of the WTV on $T_{2m}$ over the Karakoram in this season is non-significant. For air temperature changes in the lower stratosphere centered at 70 hPa, the dominant contribution from adiabatic heating is common to all seasons.

**Fig. 11:**
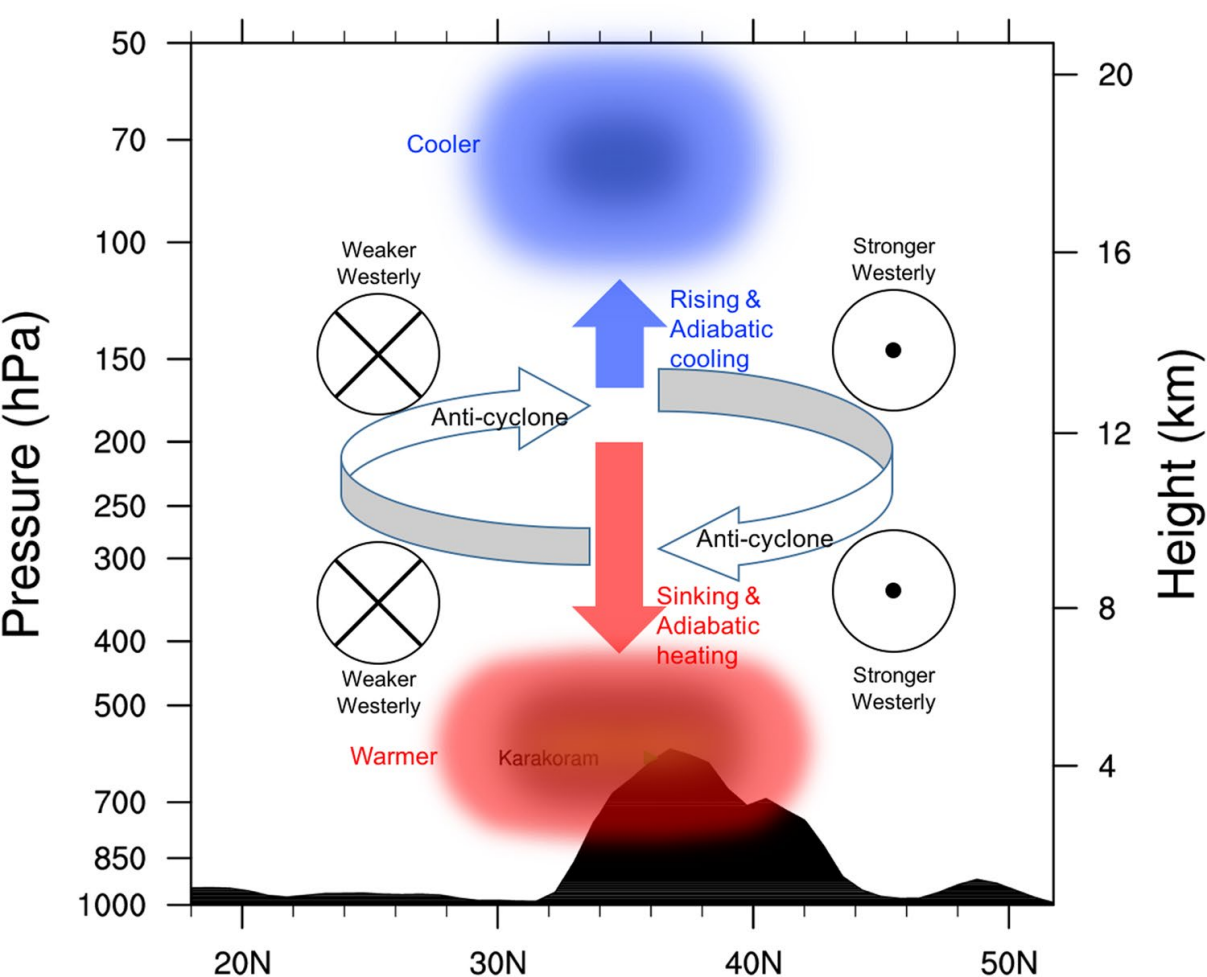
Schematic diagram of the dominant thermodynamic process (color arrow) overlapped upon the anomalous annual mean zonal wind (circle with cross or point in the middle), air temperature (color shading) and horizontal anti-cyclonic wind structure (curved 3-D arrow) for the positive KZI phase (anti-cyclonic WTV) above the western TP centered at the Karakoram

In this study, we have demonstrated through thermodynamic theoretical principles that adiabatic heating is generally the dominant mechanism through which the WTV modulates the mid-lower tropospheric and near-surface air temperature over the western TP high mountain area thus supporting the previous analytical results (FL1718). Moreover, we found that adiabatic heating is also the dominant process controlling lower stratospheric air temperature changes. This fills the knowledge gap of the impact mechanism of WTV variability on the lower stratospheric temperature response, which were described as a “high-level temperature anomaly dipole” in the initial studies (FL1718). Thus, in both the middle-to-lower troposphere and the lower stratosphere, the adiabatic heating process is the dominant mechanism through which the WTV drives air temperature variability. Figure 11 shows a schematic diagram of the dominant thermodynamic process in both the mid-low troposphere and the lower stratosphere under the anti-cyclonic WTV. The adiabatic sinking/compressing (rising/expanding) causes the air temperature to increase (decrease) in the mid-low troposphere. Meanwhile, the adiabatic rising/expanding (sinking/compressing) causes the air temperature to decrease (increase) in the lower stratosphere over the western TP high mountain areas under the anti-cyclonic (cyclonic) WTV.

It should be noted that the other two types of the major thermodynamic processes are also important in some sub-regions of the western TP. For example, the diabatic heating process (sensible heating, radiative heating, latent heating, etc.) that is effected though conduction, radiation and release is also important in summer and spring along the south slope of the TP and adjacent Indo-Gangetic plains. In summer, although the diabatic heating in the central western TP around Karakoram is not important, it positively contributes to the temperature changes over both the south slope of TP and the west tail of the TP. As the vertical velocity (Fig. 3a–d VS Fig. 5i) and total cloud cover (Figures not shown) over these two locations have opposite responses to WTV, we hypothesize that the diabatic heating effects over these two regions are attributed to different (coupled) circulation features. As a result the diabatic heating due to the cloud-radiation interaction effects over the west tail of the TP and the diabatic heating due to the latent heat release effects over the south slope of the TP are opposite in sign/phasing. Clearly though, further detailed work is required for verification of this hypothesis. Furthermore, in spring, the adiabatic heating over the near-surface of the western tail of the TP is also important in assisting the effects of the adiabatic heating. In addition, we also find that horizontal temperature advection is important over the edges of the western TP, especially at near-surface levels, which also needs further investigation. Further studies on these thermodynamic processes will be helpful in improving our understanding of the surface processes.

There are some caveats and many questions remaining. All the results above are based on ERA-Interim data. However, reanalysis data are known to have more uncertainty over high elevation areas with sparse observations (available for assimilation) than over the plains where denser observations exist. Still, ERA-Interim data has been demonstrated to better represent the thermal conditions of the atmosphere over the western TP than other reanalysis product (Forsythe et al. 2017). This means that more verification work on the thermodynamic processes over the western TP is needed using higher quality (resolution) datasets, with state-of-the-art high-resolution numerical modelling also required for further testing of the thermodynamic processes related to WTV variability. A relevant question for this is whether the thermodynamic processes related to WTV variability can be verified or simulated well by the current generation of general circulation models (GCMs).

We also show that the area with vertical motions and adiabatic heating related to WTV variability in the mid-lower troposphere and at the near-surface of the western TP is generally centred on the southwest slope of the TP in all seasons, except in summer. It seems that the mechanical forcing of the topography of the southwest slope of the TP amplifies the vertical motions and the adiabatic heating effect associated with KV/TWV variability in all seasons except for summer. We question why the thermodynamic processes related to WTV variability are different over the southwest slope of the TP. By changing the elevations of the TP topography in a GCM experiment, Yu (2009) and Yu et al. (2011a, b) were able to demonstrate that different elevation representations of TP topography induce different thermal effects over the TP in winter and summer. However, the contribution of the different elevations and aspect of the TP topography to the formation of thermodynamic processes over the western TP related to WTV variability is still an open question.

Another remaining question is how global warming may impact western TP air temperature and the WTV? Although there is a downward trend in the WTV causing near-surface air temperature changes over the western TP to be stable and even slightly cooling, resulting in glacier expansion over the Karakoram in summer (Forsythe et al. 2017), at what point will the WTV and the air temperature over the western TP be influenced by the surrounding warming globe? Additionally, as the WTV anomaly is closely related to the subtropical westerly jet in both intensity and location (FL1718), it is important to understand the interactions between the WTV and the westerly jet. Similarly, the potential impacts of changes to SST on the WTV also need further examination. For example, we know that the Eurasian atmospheric circulation is not only influenced by Atlantic SST variability (Sun et al. 2015, 2017a), but also by SST variability over the surrounding Indian Ocean and western tropical Pacific (Sun et al. 2017b, 2019). This SST variability could play an important role in the formation and development of the internal dynamics associated with the WTV. The latter three questions are now under investigation.

## Electronic supplementary material

Below is the link to the electronic supplementary material.

Supplementary material 1 (DOCX 1677 kb)
