# Supplementary material for: Thermodynamic controls of the Western Tibetan Vortex on Tibetan air temperature
Source: Clim Dyn. 2019 May 3;53(7):4267–90. doi: 10.1007/s00382-019-04785-2 (PMC6936652; doi:10.1007/s00382-019-04785-2)
Supplement: Supplementary file 1 — Supplementary material 1 (DOCX 1677 kb) [file 382_2019_4785_MOESM1_ESM.docx]

**Thermodynamic Controls of the Western Tibetan Vortex on Tibetan Air Temperature**

Xiao-Feng Li^[[1]](#footnote-1)^*, Hayley J. Fowler, Jingjing Yu, Nathan Forsythe, Stephen Blenkinsop, David Pritchard

*School of Engineering, Newcastle University, Newcastle upon Tyne NE1 7RU, UK*

Prepared for *Climate Dynamics*

**Supplementary Information**

1. **Climatological mean circulations**

The western TP is located in subtropics under the Subtropical Westerly Jet (SWJ), where the westerlies prevail all year round (see **Figure S1**), but the wind speed over the western TP is much lower than over its neighboring area. **Figure S1** shows that the total wind speed ($\sqrt{\left( u^{2}+v^{2} \right)/2}$, $u$ and $v$ are zonal and meridional wind components) in the central location of the western TP near the Karakoram is much lower than for either the north or south side of the western TP in all four seasons. This suggests the atmospheric mass and energy exchange rate between the central western TP and surrounding areas by horizontal advection is weaker than that for the north and south of the western TP, i.e. the air temperature in the central western TP is influenced less by horizontal temperature advection than that over the areas for the north and south of the western TP.


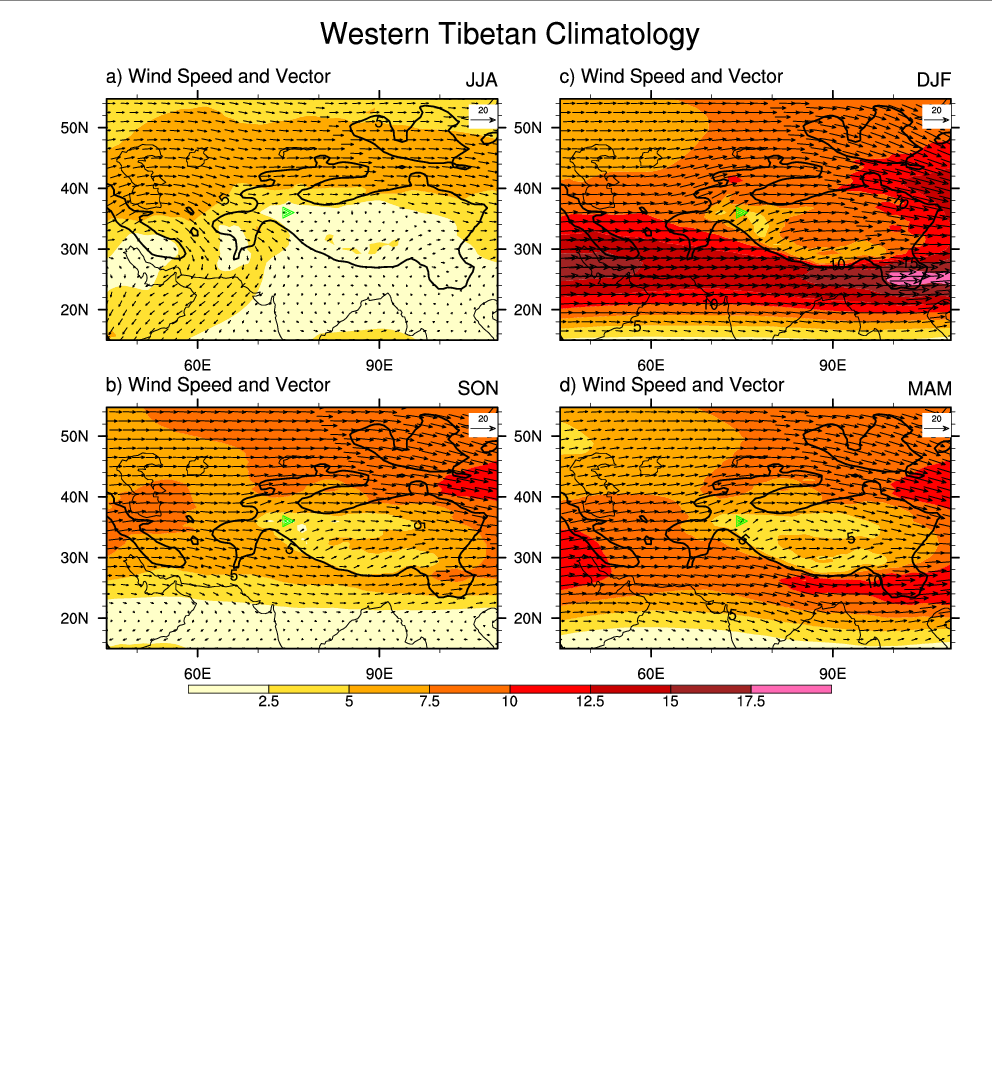


- 1. **Figure S1** Climatological mean horizontal wind vectors (m s^-1^) and absolute wind speed (colour-shaded, m/s) at the 500-hPa level (the near surface of the western TP) over the TP for a) summer (JJA), b) autumn (SON), c) winter (DJF) and spring (MAM) seasons in 1979-2016. Wind vector length scale is in the upper right corner of panels; the bold black lines denote topography above 1,500 m. The green triangle denotes the central position (36°N, 75°E) of the Karakoram focus area.

- 1. **Figure S2** Vertical profile of the climatological mean zonal wind (m/s, color shading and contours) at the upper-stream of Tibetan Plateau (0~60°E) in a) summer (JJA), b) autumn (SON), c) winter (DJF) and spring (MAM) seasons in 1979-2016. The real, dashed and bold real grey contour lines denote the positive, negative and zero values of the zonal wind, and the contour interval is 5 m/s. The black shading area denotes TP topography along the 75°E.

In contrast to the central western TP, the total wind speed is much stronger in neighboring areas to the south and north, which is largely due to the prevailing westerlies. In summer (**Figure S1a**), the westerlies to the north side of the western TP are stronger than those to the south side at the near-surface (500-hPa level), which is due to the axis of the SWJ being located to the north side of the western TP (not shown). In winter, in contrast, the westerlies to the south side of the western TP are stronger than those to the north side (**Figure S1c**) at the near-surface as the axis of the SWJ moves to the south side of the western TP (not shown) associated with the solar altitude changes. During autumn (SON) and spring (MAM), the westerlies to north and south side of the western TP are relatively comparable. The change in relative intensity of the westerlies to the north and south of the western TP coincides with the seasonal changes of the Karakoram Zonal Sheer (KZS) found in Forsythe et al. (2017) and Li et al. (2018).

# Meridional distribution of contributions in four seasons

The meridionally-averaged difference of the three major TEE terms over the main body of the TP (24.75°N ~44.5°N) are computed to show the general geological distributions of the contributions from major thermodynamic processes. Shown as Figure S3, the meridionally-averaged $\delta\left( ADH \right)$ is relatively uniform and positive over the TP west of 90°E, but it is either with a much smaller value (or magnitude) over the TP east of 90°E (figures not shown) than does over the western TP or is highly disturbing between positive and negative values within very short east-west distance over the eastern TP. For example, in summer, the magnitudes of the meridionally-averaged $\delta\left( ADH \right)$ are smaller over the eastern TP than over the western TP; in autumn and spring, the meridionally-averaged $\delta\left( ADH \right)$ generally reverts into negative, opposing to the positive $\delta\left( ADH \right)$ over the western TP; in winter, the magnitude of the positive meridionally-averaged $\delta\left( ADH \right)$ over the western TP is uniform and extend to over eastern TP, except that it shows a relatively smaller magnitude and with some negative disturbance centering at 100°E over the eastern TP, which is consistent with the fact that the horizontal structure of the WTV is extended from the western TP to the eastern TP in winter (FL78). So, the contributions of the adiabatic rising/sinking process under the WTV variability is important and thriving over the western TP, but is relatively non-stable with small magnitude over the eastern TP. This is reasonable as the main body of the WTV is located over the western TP rather than over the eastern TP (FL78).

Actually, the positive contribution from the adiabatic sinking/rising processes is the dominant factor of the air temperature changes in central western TP in summer and in nearly entire western TP in other three seasons. This is demonstrated well on 300 hPa, the central level with greatest air temperature changes caused by the WTV variability in the mid-lower troposphere. Shown as Figure 7a, the summertime $\delta\left( ADH \right)$ (red dashed line) at the 300-hPa level over the central western TP near to 70°E reaches above 1.2 K day^-1^, balancing the sum of contributions from horizontal advection (green solid line) and diabatic heating (blue solid line), suggesting the adiabatic sinking compressing (rising expanding) process over the main body centering 70°E of the western TP is the dominant process inducing the air temperature increases (decreases) in the mid-lower troposphere under the anti-cyclonic (cyclonic) WTV. In other three seasons (Figure 7ceg), the $\delta\left( ADH \right)$ is generally the single positive term that balances the sum of other two negative terms ($\delta\left( HTAD \right)$ and $\delta\left( DH \right)$) above most parts of the TP west of 90°E, demonstrating the overwhelming contribution of the the adiabatic sinking compressing (rising expanding) process to the temperature increases (decreases) over western TP in the mid-lower troposphere under the anti-cyclonic (cyclonic) WTV. Similarly, on 500 hPa (Figure 7bdfh), the near surface level of the western TP, the adiabatic sinking/rising processes is still the single positive term in central western TP in summer and in nearly all western TP in other seasons, although there are minor differences, for example the horizontal temperature advection centering at 72°E in autumn (Figure 7) becomes dominant at the 500 hPa level, and etc.

Further analysis in the fowling four sections will present the full geological distribution of the contributions of the three major TEE terms to the air temperature changes under the WTV variability season by season.

**Figure S3** Meridionally-average (24.75°N ~44.5°N) of the $\delta\left( ADH \right), \delta\left( HTAD \right)$ and $\delta\left( DH \right)$ (units: K Day^-1^) in a-b) summer (JJA), b-c) autumn (SON), d-e) winter (DJF) and f-h) spring (MAM) at multiple levels above the Tibetan Plateau as a function of longitude for summer (JJA) in 1979-2016. Upper row is for 300 hPa, bottom row is for 500 hPa. The topography of Tibetan Plateau is shown in the bottom of each panel (24.75°N ~44.5°N, 60°E~90°E), the grey shaded area denotes the topography above 1,500m. Values below 1,500 m were masked out before the averaging. Black dot denotes the central position (36°N, 75°E) of Karakoram focus area.$Type equation here.$

# Distribution of the total cloud cover in four seasons

The difference of the composited total cloud cover (TCC), $\delta\left( TCC \right)$, is shown as the Figure S4. In summer (Figure S4a), the significant negative $\delta\left( TCC \right)$ is observed over most of the TP including the western TP, and the significant positive $\delta\left( TCC \right)$ is observed over the southwest slope of the TP. It means the TCC is decreased (increased) over most of the TP including the western TP, but increased (decreased) over the southwest slope of the TP including the Central Himalaya (far Northern India, western Nepal) under the anti-cyclonic (cyclonic) WTV; and the change of the TCC over the western TP and that over the southwest slope of the TP is opposite and is shown as a “dipole-like” structure. This marches well with the “diplo-like” structure in $\delta\left( w \right)$ (Figure S4a VS Figure 3a-d), because the anomalous sinking motions are basically responsible for less cloudiness, and vice versa. In other three seasons (Figure S4bcd), the significant negative $\delta\left( TCC \right)$ is mainly observed over the western TP including the southwest slope of the TP, matching with the significant negative $\delta\left( w \right)$ (Figure S4bcd VS Figure 3e-f and Figure 4). Specially, the “diplo-like” structure in springtime $\delta\left( TCC \right)$ is much weaker than that in $\delta\left( w \right)$ (Figure 4e-h), as the positive changes of the $\delta\left( TCC \right)$ over southwest slope are very weaker and not significant at 0.05 level.


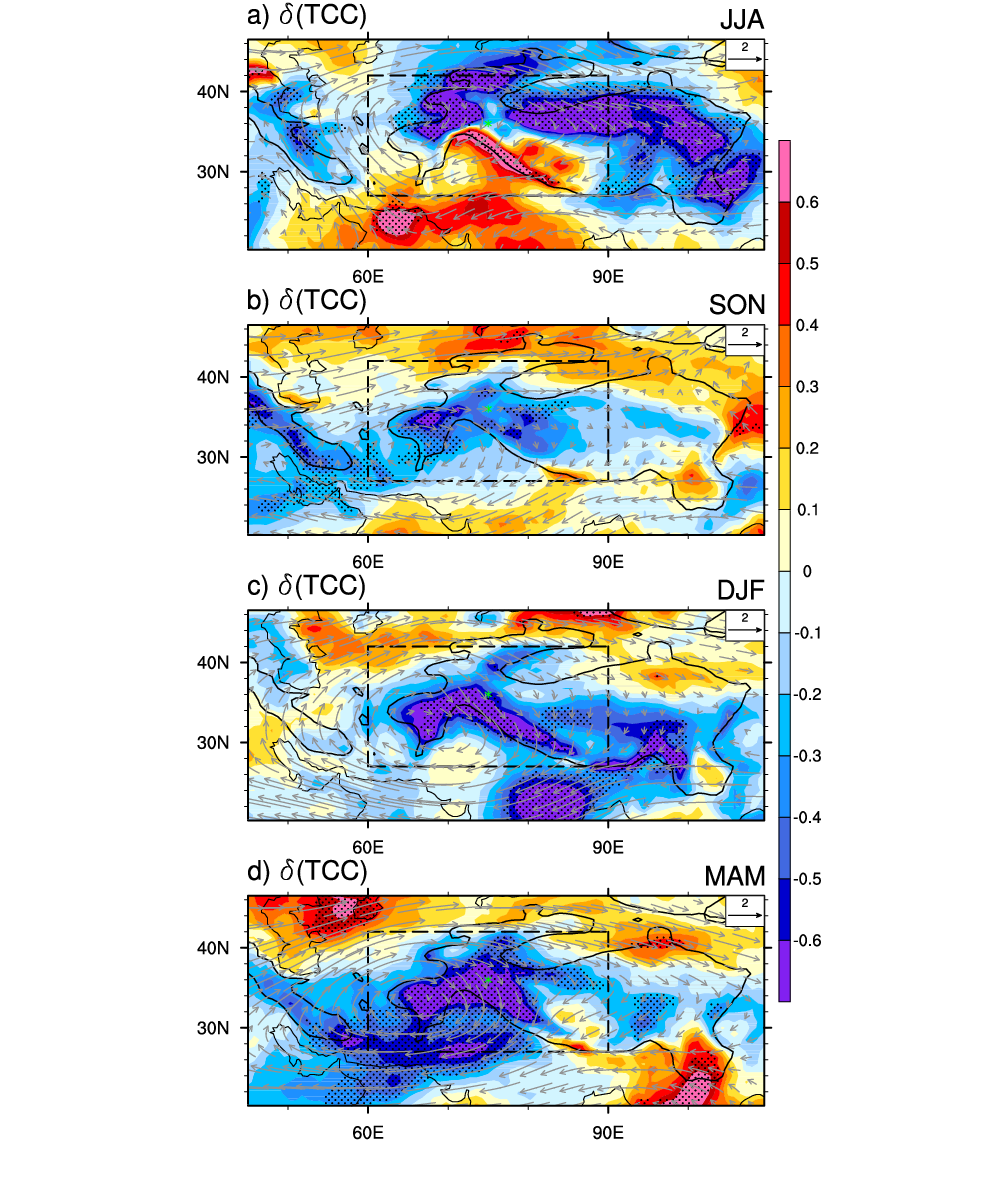


**Figure S4** The difference of the total cloud cover (TCC) ($\delta\left( TCC \right)$, color shading, %) 500-hPa wind vectors (grey vectors, m s^-1^) between positive and negative KZI events (positive minus negative phase) in a) summer (JJA), b) autumn (SON), c) winter (DJF) and d) spring (MAM) for 1979-2016. The black dots denote significance of the TCC above the 0.05 level, after taking account of the efficient number of degrees of freedom (Zar 1984; Li et al. 2013). Bold-black-outlie denotes topography above 1,500 m. Grey shaded area denotes the topography above the isobaric surface. The green star denotes the central Karakoram (36° N, 75° E). The dash rectangle denotes the western TP focus area (within this domain above 1,500 m is defined as the western TP area).

### REFERENCES

Forsythe, N., H. J. Fowler, X.-F. Li, S. Blenkinsop, and D. Pritchard, 2017: Karakoram temperature and glacial melt driven by regional atmospheric circulation variability. *Nature Climate Change*.

Li, X.-F., H. J. Fowler, N. Forsythe, S. Blenkinsop, and D. Pritchard, 2018: The Karakoram Vortex/Western Tibetan Vortex: Seasonal and Inter-Annual variability. *Climate Dyn.*, **in press.** Doi: 10.1007/s00382-018-4118-2.

Zwiers, F. W., and H. von Storch, 1995: Taking Serial Correlation into Account in Tests of the Mean. *J. Climate*, **8,** 336-351.

1. * ***Corresponding author address***: Dr. Xiao-Feng Li, School of Engineering, Newcastle University, Newcastle upon Tyne NE1 7RU, UK

   E-mail: Xiaofeng.Li@newcastle.ac.uk [↑](#footnote-ref-1)
